# The GATA transcription factor GNC plays an important role in photosynthesis and growth in poplar

**DOI:** 10.1093/jxb/erz564

**Published:** 2019-12-24

**Authors:** Yi An, Yangyan Zhou, Xiao Han, Chao Shen, Shu Wang, Chao Liu, Weilun Yin, Xinli Xia

**Affiliations:** 1 Beijing Advanced Innovation Center for Tree Breeding by Molecular Design, College of Biological Sciences and Technology, National Engineering Laboratory of Tree Breeding, Beijing Forestry University, Beijing, China; 2 State Key Laboratory of Subtropical Silviculture, School of Forestry and Biotechnology, Zhejiang A&F University, Lin’an, Hangzhou, China; 3 University of Essex, UK

**Keywords:** CRISPR/Cas9, fast growth, GATA transcription factor, nitrogen, photosynthesis, poplar

## Abstract

GATA transcription factors are involved in the regulation of diverse growth processes and environmental responses in Arabidopsis and rice. In this study, we conducted a comprehensive bioinformatic survey of the GATA family in the woody perennial *Populus trichocarpa*. Thirty-nine *Populus GATA* genes were classified into four subfamilies based on gene structure and phylogenetic relationships. Predicted *cis*-elements suggested potential roles of poplar *GATA* genes in light, phytohormone, development, and stress responses. A poplar GATA gene, *PdGATA19*/*PdGNC* (GATA nitrate-inducible carbon-metabolism-involved), was identified from a fast growing poplar clone. *PdGNC* expression was significantly up-regulated in leaves under both high (50 mM) and low (0.2 mM) nitrate concentrations. The CRISPR/Cas9-mediated mutant *crispr-GNC* showed severely retarded growth and enhanced secondary xylem differentiation. *PdGNC*-overexpressing transformants exhibited 25–30% faster growth, 20–28% higher biomass accumulation, and ~25% increase in chlorophyll content, photosynthetic rate, and plant height, compared with the wild type. Transcriptomic analysis showed that *PdGNC* was involved in photosynthetic electron transfer and carbon assimilation in the leaf, cell division and carbohydrate utilization in the stem, and nitrogen uptake in the root. These data indicated that *PdGNC* plays a crucial role in plant growth and is potentially useful in tree molecular breeding.

## Teaser

Thirty-nine GATA transcription factor genes were identified in the poplar genome. The GATA transcription factor PdGNC positively regulates photosynthesis and plant growth by promoting chloroplast development in poplar.

## Introduction

Poplar is an important woody model plant that is distinguished from annual herbaceous model plants, such as Arabidopsis and rice (*Oryza sativa*), on account of its woody secondary growth and perennial habit. The rapid-growth characteristic of *Populus* spp. renders the wood commercially valued for paper, timber, construction materials, and biofuel production (Jansson and Douglas, 2007; Polle *et al.*, 2013). Many factors affect the growth rate of trees, including photosynthetic efficiency, nutrient utilization, and hormone stimulation (Euring *et al.*, 2014; Lu *et al.*, 2015). Photosynthesis, as the most important process for carbon fixation and metabolism, converts light energy into chemical energy for plant development and growth (Yamori and Shikanai, 2016). Hence, chloroplast architecture and composition have a marked influence on photosynthetic capability, and a high chlorophyll content contributes to increased light-harvesting capacity. Efficient photosystems fix higher quantities of carbon and produce greater amounts of carbohydrates, which are requisite for plant growth and biomass accumulation.

Nitrogen (N) is an essential macronutrient for plant growth as a component of many vital molecules, such as nucleic acids, amino acids, proteins, and certain plant hormones. Sufficient N availability is necessary for plant growth and development, whereas N starvation or excess can expose plants to N stress (Yang *et al.*, 2015). Nitrogen stress has marked effects on N uptake and metabolism (Luo *et al.*, 2015), chlorophyll synthesis (An *et al.*, 2014), lignin content (Rueda-López *et al.*, 2015), and plant biomass accumulation (Hudson *et al.*, 2013). Nitrogen-deficient plants exhibit low net photosynthetic rates and reduced biomass production (Bondada and Syvertsen, 2003; Nunes-Nesi *et al.*, 2010). Carbon (C) and N are integral components of plants, and C and N metabolic processes are closely linked (Coruzzi and Bush, 2001). The N level *in planta* affects photosynthesis and C assimilation, whilst C metabolites contribute to N absorption and utilization. Thus, regulation of C and N interaction is crucial for modification of plant development and growth.

GATA proteins are evolutionarily conserved transcription factors that interact with the WGATAR (W=T or A; R=G or A) sequence motif and are ubiquitous in eukaryotes, including fungi, metazoans, and plants (Lowry and Atchley, 2000; Scazzocchio, 2000; Patient and McGhee, 2002; Teakle *et al.*, 2002; Reyes *et al.*, 2004; An *et al.*, 2014). In Arabidopsis, a majority of the previously classified 30 GATA proteins contain only one zinc finger, C-X_2_-C-X_18_-C-X_2_-C, but a few proteins contain two zinc fingers, C-X_2_-C-X_20_-C-X_2_-C (Reyes *et al.*, 2004; Bi *et al.*, 2005). Previous studies have revealed that GATA transcription factors are widely involved in regulation of plant developmental and growth processes, such as seed germination (Liu *et al.*, 2005), chloroplast development (Bi *et al.*, 2005; Chiang *et al.*, 2012; An *et al.*, 2014, Zhang *et al.*, 2015), flower development (Mara and Irish, 2008), response to light (Luo *et al.*, 2010), and lateral root initiation identity (De Rybel *et al.*, 2010). In addition, some studies indicate that GATA factors play roles in N metabolism (Reyes *et al.*, 2004; Bi *et al.*, 2005; Hudson *et al.*, 2011; An *et al.*, 2014). GATA-binding motifs have been detected in regulatory regions of many genes involved in N assimilation, such as nitrate reductase, nitrite reductase, and glutamine synthetase (Jarai *et al.*, 1992; Oliveira and Coruzzi, 1999; Hudson *et al.*, 2011). Thus, GATA transcription factors play a potential role in coordination of nutrition utility and vegetative growth.

The clustered regularly interspaced short palindromic repeats (CRISPR) system is a powerful tool in plant genome engineering and has been used successfully for genome editing of the woody plant *Populus* (Fan *et al.*, 2015; Zhou *et al.*, 2015; Wang *et al.*, 2017; Xu *et al.*, 2017). In this study, we conducted a genome-wide survey of *Populus trichocarpa* GATA-related sequences and explored their expression in different tissues in response to three nitrate concentrations. The poplar GATA family member, *GNC* (GATA Nitrate-inducible Carbon-metabolism-involved), was significantly induced by N and is an ortholog of Arabidopsis *AtGNC* (An *et al.*, 2014). To extend our previous study of *Populus GNC* in Arabidopsis, we employed the CRISPR/Cas9 system to produce a *GNC* knockout mutant and overexpressed *GNC* in *Populus* to examine functions of *GNC* in the growth and development of a woody plant.

## Materials and methods

### Identification of GATA transcription factors in poplar

BLAST and keyword searches were utilized to compile a comprehensive and non-redundant data set of *Populus trichocarpa* proteins containing the conserved GATA domain. The query proteins and nucleotide sequences were obtained from different resources: chicken GATA1 (AAA49055), *Aspergillus nidulans* AreA (P17429), *Neurospora crassa* WC1 (Q01371), and all 30 documented GATA family genes of Arabidopsis (Reyes *et al.*, 2004). A keyword search was performed in Phytozome (http://www.phytozome.net) for putative GATA factors by searching ontologies with the term of the GATA domain (PF00320). All sequences had *e*-values below 10^−6^. Sequences with a length of more than 100 amino acids were selected for further analysis. The candidate sequences were analysed with SMART (Letunic *et al.*, 2012), InterPro (http://www.ebi.ac.uk/interpro/), and Pfam (http://pfam.xfam.org) software. The results were compared against predicted members of the GATA family in the PlantTFDB (Jin *et al.*, 2014) and PlnTFDB databases (Pérez-Rodríguez *et al.*, 2010). Paralogous pairs were explored using the Plant Genome Duplication Database (http://chibba.agtec.uga.edu/duplication/). A *cis*-acting element analysis of the promoter region (2 kb of genomic DNA sequence upstream from the translation start site) was conducted with the PlantCARE database (http://bioinformatics.psb.ugent.be/webtools/plantcare/html/).

### Multiple sequence alignment and phylogenetic analysis

For exon–intron structural analysis, the genomic sequence and protein sequence for each putative poplar *GATA* gene were downloaded from Phytozome. The gene structure was analysed using the Gene Structure Display Server tool (Guo *et al.*, 2007). Conserved amino acids were viewed using Logo (http://weblogo.berkeley.edu/logo.cgi). A phylogenetic tree derived from alignment of *Populus* GATA family amino acid sequences was constructed using Phylogeny (http://www.phylogeny.fr). The chromosomal location of the poplar *GATA* genes was determined using PopGenIE (http://www.popgenie.org/). An unrooted tree was constructed using MEGA 6 employing the neighbor-joining method and support for the tree topology was assessed by performing a bootstrap analysis with 1000 replicates (Tamura *et al.*, 2013). Core consensus sequence logos of Arabidopsis and poplar *GATA* genes were created using Weblogo (http://weblogo.berkeley.edu/).

### Plant materials and growth conditions

Cuttings of *Populus* clone NE-19 (*P. nigra* × (*P. deltoides* × *P. nigra*)) were cultivated following the description of Hao *et al.* (2011). Plants were pre-cultured in buckets containing sufficient nitrate medium (SN; 10 mM NO_3_^−^) for 1 week and then transplanted into buckets containing low nitrate medium (LN; 0.2 mM NO_3_^−^) or high nitrate medium (HN; 50 mM NO_3_^−^). Plants cultured in SN were considered to be the control. The hydroponic culture media were maintained at pH 5.8–6.0 and refreshed at weekly intervals. The growth chamber was set to 16 h white light (07.00–23.00 h) at 24 °C and 8 h darkness (23.00–07.00 h) at 20 °C, with 150 μmol m^−2^ s^−1^ irradiation.

### RNA extraction and transcript analysis

Leaf, stem, and root samples taken from *Populus* clone NE-19 plants treated for 1 week with the three nitrate concentrations, and from different *Populus GNC* modified plants and then immediately frozen in liquid nitrogen and stored at −80 °C until RNA extraction. Three replications consisting of nine seedlings for each sampling time point were used. Total RNA was isolated using the RN38 EASYspin Plus Plant RNA Kit (Aidlab Biotech, Beijing, China). First-strand cDNA synthesis was performed using first-strand M-MLV Reverse Transcriptase and an oligo(dT) primer (Promega, Madison, WI, USA) following the manufacturer’s instructions. Samples were diluted 10 times (~100 ng μl^−1^) prior to quantitative real-time PCR (qPCR) analysis. The specific primers used to quantify *Populus* NE-19 *GATA* genes, *PdGATA* genes, and differentially expressed genes by qPCR were designed according to the corresponding gene sequences in the *Populus trichocarpa* reference genome (see Supplementary Tables S1, S2 at *JXB* online). The PCR mixture consisted of 1 μl sample, 0.6 μl (10 μM) forward primer, 0.6 μl (10 μM) reverse primer, 5.8 μl RNase-free ddH_2_O, 10 μl SuperReal PreMix Plus, and 2 μl ROX Reference Dye in a total volume of 20 μl. The reaction was amplified for 40 cycles at 95 °C for 10 s, 55 °C for 30 s, and 72 °C for 32 s.

### *PdGNC* gene cloning and transformation

The *Populus GATA* gene *PdGNC* (GenBank accession KF541241), an ortholog of *PtrGATA19* (Potri.006G229200), was identified and characterized from *Populus* clone NE-19. Extraction of total RNA and subsequent cDNA synthesis from leaves of *Populus* were performed using the aforementioned methods. The *PdGNC* cDNA sequence was amplified using the primers PdGNC-F (GGCCCTTTTAGCCTTGTTGTTTGT) and PdGNC-R (TCAGCTGTGAATAAAGCCACAAG). The 35S promoter-driven overexpression cassette *35S:PdGNC* was constructed by introducing the *PdGNC* coding sequence into the pCAMBIA1301 expression vector and then transformed into *Agrobacterium tumefaciens* strain GV3101. 

For *Agrobacterium*-mediated *35S:PdGNC* transformation of *Populus* clone 717, *Agrobacterium*-mediated *35S:PdGNC* cells were collected and resuspended to OD_600_=0.3–0.4. The leaf discs were soaked for 1 h on a shaker with the resuspended cells at room temperature. The inoculated leaf discs were co-cultivated at 19–25 °C in the dark for 2–3 d. The leaf discs were washed with double-distilled water and cultured on medium for callus inducement supplemented with 500 mg l^−1^ cefotaxime and 50 mg l^−1^ kanamycin for 10–30 d in the dark. Shoot and root regeneration from the calli was induced on medium supplemented with 100 mg l^−1^ kanamycin for several weeks to months. The total period from co-cultivation to regeneration of a rooted transgenic plantlet varied widely among clones, ranging from about 4 to 8 months (Han *et al.*, 2000). Transgenic lines were selected by kanamycin and identified by qPCR. The positive T_0_ transgenic plants were regenerated on antibiotic-free media that were also used for the controls to ensure their synchronization under the growth conditions for subsequent experimental analysis. Three transgenic lines chosen for further analysis showed higher expression levels of the target gene (see Supplementary Fig. S1).

### CRISPR/Cas9-mediated targeted mutagenesis of *Populus GNC*

The single-guide RNA (sgRNA) sequence for *Populus GNC* of clone 717 was designed based on the SNP-bearing *Populus* 717 genomic database (Zhou *et al.*, 2015). The target site of the designed sgRNA was confirmed by amplification and sequencing (the primers are listed in Supplementary Table S3). The designed sgRNA was assembled into the entry vector pEn-Chimera and then an expression construct was generated with the destination vector pDe-CAS9 (Fauser *et al.*, 2014), using Gateway recombination cloning technology (Thermo Fisher Scientific, Waltham, MA, USA). The CRISPR/Cas9 construct was transformed into *Populus* clone 717 using the aforementioned method. For confirmation of positive transgenic plants, we used the primers GNC_Mut_F1 and GNC_Mut_R1 to amplify the genomic region flanking the target sites (see Supplementary Table S3). The forward primer was located 379 bp upstream of the target and the reverse primer was located 221 bp downstream. Individual amplicons from each transgenic event were visualized in agarose gel. Bands were excised using a clean razor and DNA was extracted using the TIANgel Midi Purification Kit (Tiangen, Beijing, China). Sanger DNA sequencing of PCR amplicons was used to evaluate the editing conditions of CRISPR/Cas9 transfection. Individual sequences were aligned to the wild type (WT) sequence using SnapGene to determine the severity of the mutation on the predicted final peptide sequence (see Results Fig. 3C and Supplementary Fig. S6).

**Fig. 1.**
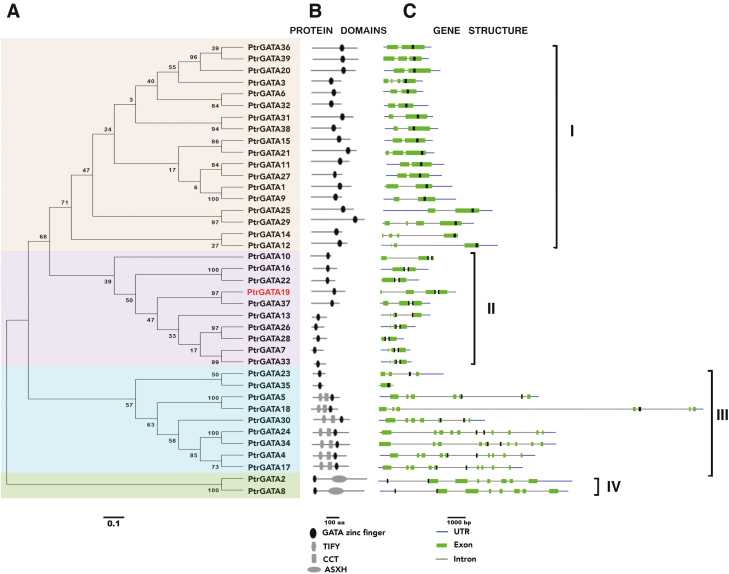
Phylogenetic analysis of GATA family genes in *Populus*. (A) Neighbor-joining tree constructed from full-length amino acid sequences from *Populus* GATA genes. (B) Protein domain distribution of *PtrGATA* members. (C) Gene structure analysis of *PtrGATA* genes. The position of nucleotide sequences coding for the GATA zinc finger is depicted in black.

**Fig. 2.**
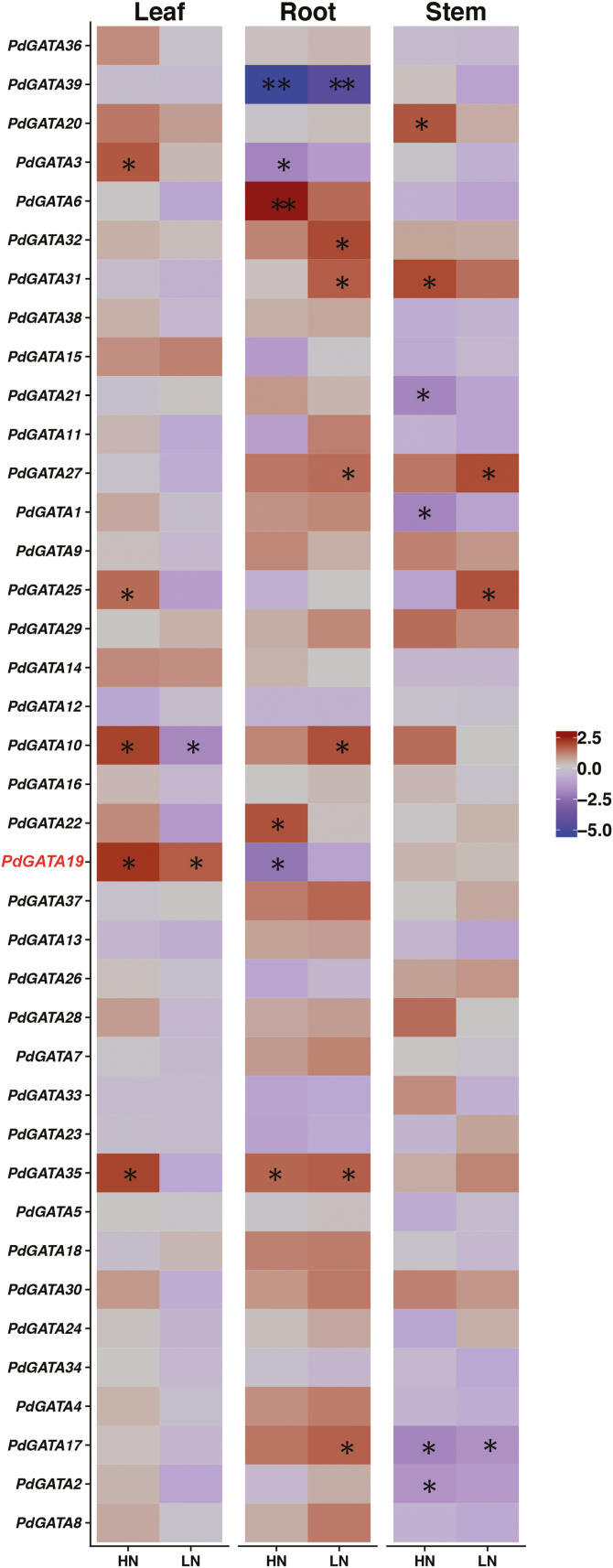
Expression profiles of *GATA* genes in the leaf, root, and stem of *Populus* NE-19 under high nitrate (HN), sufficient nitrate (SN), and low nitrate (LN) for 1 week. The expression levels of genes were determined using qPCR and normalized by log2 transformation. The data are the mean ±SD (*n*=3 experiments). Each experiment comprised three biological replicates. Asterisks indicate a significant difference (*P<0.05, **P<0.01; one-way ANOVA).

### Physiological measurements and histological analysis

Chlorophyll content was measured using leaf samples (~1 g) from 8-week-old plants. Leaves were immersed in 1 ml *N*,*N*-dimethylformamide overnight at 4 °C. Chlorophyll *a* and chlorophyll *b* contents were determined spectrophotometrically using published equations (Porra *et al.*, 1989). The Li-6400 Portable Photosynthesis System (Li-Cor, Lincoln, NE, USA) was used to determine photosynthetic rates. Mature, fully expanded leaves of 8-week-old plants were used for measurements under an ambient CO_2_ concentration of 360 μmol mol^−1^, photosynthetic photon flux density of 600 μmol m^−2^ s^−1^, and chamber temperature of 24 °C at 10.00–11.00 h.

Preprocessing of samples for transmission electron microscopy (TEM) followed the method of An *et al.* (2014). The same position in the central portion of leaves was fixed by vacuum infiltration in 2.5% (v/v) glutaraldehyde and 0.2 M sodium phosphate buffer (pH 7.2). The samples were incubated in three changes of the fixative for 15 min each. Samples were post-fixed in 2% (w/v) OsO_4_ overnight. All TEM images were captured at 100 kV on a TEM 1010 apparatus (JEOL, Tokyo, Japan) equipped with an XR-41B AMT digital camera (Advanced Microscopy Techniques, Woburn, MA, USA). Transverse sections (50 μm thickness) were prepared using a Vibratome (Series 1000, Heath Company, USA), stained with toluidine blue, and visualized with a Leica microscope (Gerttula *et al.*, 2015).

### RNA-sequencing and gene co-expression network analysis

Leaf, stem, and root samples from WT, *oxPdGNC*, and *crispr-GNC* plants were harvested. Three biological replicates were included for each tissue and in total 27 samples were prepared for 150 bp paired-end sequencing using an Illumina HiSeq X Ten platform. Library construction and sequencing were performed following the manufacturer’s protocols. The data were trimmed and mapped to the *Populus tremula* × *P. alba* 717-1B4 reference genome (http://aspendb.uga.edu/index.php/databases/spta-717-genome). Differentially expressed genes were detected using Cufflinks software following the criteria (|log_2_(fold-change)| >1 and false discovery rate <0.05). Gene ontology (GO) enrichment analysis was performed using the R packages GOstats and GSEABase. The gene expression abundance was calculated and used for weighted gene co-expression network analysis (WGCNA; Langfelder and Horvath, 2008). The soft threshold power of the adjacency matrix for the co-expression relationship between genes was 8. Hierarchical clustering was performed with a minimum module size of 400 and a cut height of 0.994. Different colors were assigned to different modules. The RNA-sequencing data were deposited in the National Center of Biotechnology Information Sequence Read Archive database under BioProject PRJNA449161.

## Results

### Identification and analysis of poplar GATA transcription factors

We identified a total of 39 unique putative *Populus GATA* genes in the published *Populus trichocarpa* reference genome and consecutively designated the genes *PtrGATA1* to *PtrGATA39* based on their genomic location (Table 1). The GATA family genes were distributed on 15 of the 19 chromosomes at various densities except *PtrGATA39*, which was located in an as-yet-unattributed scaffold 694 (see Supplementary Fig. S2). The most recent *Populus* genome-wide duplication event contributed to gene duplication and expansion of gene families (Tuskan *et al.*, 2006). We identified 10 paralogous pairs located in the segmental duplicated blocks (Supplementary Fig. S2), whereas 19 genes located in these blocks did not have a corresponding paired gene, which suggested that GATA family genes experienced diverse evolutionary processes after chromosome duplication.

**Table 1.** Summary of *Populus* GATA transcription factors

Name	Gene model ID	Pfam ID	gDNA	Transcript	CDS	Domains	AA	MM	GRAVY	p*I*	Homologs in Arabidopsis
PtrGATA1	Potri.001G053500	PF00320	2425	1801	1011	257–292	336	38.29	−0.818	9.68	At3g24050 (AtGATA1)
PtrGATA2	Potri.001G151700	PF00320	6921	2837	1659	15–50	552	61.30	−0.655	6.96	At4g17570 (AtGATA26)
PtrGATA3	Potri.001G188500	PF00320	1336	1000	753	166–201	250	28.01	−0.617	5.41	At4g32890 (AtGATA9)
PtrGATA4	Potri.002G110800	PF06200, PF06203, PF00320	2190	882	366	213–250	360	39.28	−0.72	4.87	At3g21175 (AtGATA24)
PtrGATA5	Potri.002G110900	PF06200, PF06203, PF00320	5321	1513	873	201–237	290	31.47	−0.628	5.93	At4g24470 (AtGATA25)
PtrGATA6	Potri.002G142800	PF00320	1357	1212	741	163–198	246	27.39	−0.738	6.03	At2g45050 (AtGATA2)
PtrGATA7	Potri.002G199800	PF00320	1112	894	444	30–65	147	16.6	−0.879	9.4	At5g49300 (AtGATA16)
PtrGATA8	Potri.003G082800	PF00320, PF04683	6299	2462	1623	7–42	540	60.24	−0.672	6.58	At4g17570 (AtGATA26)
PtrGATA9	Potri.003G174800	PF00320	2453	1564	780	179–213	259	29.02	−0.797	9.17	At3g24050 (AtGATA1)
PtrGATA10	Potri.003G213300	PF00320	1956	681	226	180–212	226	25.42	−1.071	8.72	At3g20750 (AtGATA29)
PtrGATA11	Potri.004G161500	PF00320	1957	1753	984	246–281	327	36.30	−0.724	8.53	At5g66320 (AtGATA5)
PtrGATA12	Potri.004G211800	PF00320	4420	1926	906	236–271	301	33.97	−0.675	8.57	At1g08010 (AtGATA11)
PtrGATA13	Potri.005G020500	PF00320	1853	1182	486	26–61	161	17.59	−0.675	9.78	At5g49300 (AtGATA16)
PtrGATA14	Potri.005G066100	PF00320	2822	771	771	190–225	256	29.32	−0.559	8.75	At4g36240 (AtGATA7)
PtrGATA15	Potri.005G117600	PF00320	1640	1403	1002	249–284	333	36.84	−0.630	7.10	At5g66320 (AtGATA5)
PtrGATA16	Potri.005G122700	PF00320	1176	1672	765	137–172	254	28.60	−0.957	7.55	At3g50870 (AtGATA18)
PtrGATA17	Potri.005G152500	PF06200, PF06203, PF00320	5157	1684	1098	218–255	365	39.71	−0.626	5.13	At1g51600 (AtGATA28)
PtrGATA18	Potri.005G152800	PF06200, PF06203, PF00320	12075	1150	867	200–237	288	31.46	−0.698	5.67	At4g24470 (AtGATA25)
PtrGATA19	Potri.006G229200	PF00320	2820	1396	1068	222–257	355	39.45	−0.610	9.04	At5g56560 (AtGATA21)
PtrGATA20	Potri.006G237700	PF00320	1914	1775	1122	262–297	373	41.40	−0.791	6.04	At5g25830 (AtGATA12)
PtrGATA21	Potri.007G016600	PF00320	1676	1453	1131	292–372	376	41.81	−0.589	6.95	At5g66320 (AtGATA5)
PtrGATA22	Potri.007G024500	PF00320	1438	1304	765	138–173	254	28.67	−0.941	8.46	At3g50870 (AtGATA18)
PtrGATA23	Potri.007G116600	PF06200, PF06203, PF00320	2108	1255	429	53–90	142	14.88	−0.318	8.47	At4g24470 (AtGATA25)
PtrGATA24	Potri.007G116700	PF00320	5875	1636	1155	218–255	384	43.22	−0.852	4.90	At3g21175 (AtGATA24)
PtrGATA25	Potri.008G038900	PF00320	3709	1909	1065	258–302	354	38.94	−0.597	6.52	At4g32890 (AtGATA9)
PtrGATA26	Potri.008G213900	PF00320	1290	1091	417	29–64	138	14.78	−0.909	9.69	At3g06740 (AtGATA15)
PtrGATA27	Potri.009G123400	PF00320	1898	1691	990	248–283	329	36.59	−0.629	6.61	At5g66320 (AtGATA5)
PtrGATA28	Potri.010G001300	PF00320	867	771	462	34–69	153	16.42	−0.690	9.85	At3g06740 (AtGATA15)
PtrGATA29	Potri.010G223300	PF00320	3408	1885	1341	350–385	446	49.07	−0.641	5.93	At4g32890 (AtGATA9)
PtrGATA30	Potri.010G251600	PF06200, PF06203, PF00320	3543	1641	924	231–268	307	33.37	−0.893	7.80	At1g51600 (AtGATA28)
PtrGATA31	Potri.013G059600	PF00320	1642	1288	888	227–262	295	32.67	−0.746	6.53	At5g25830 (AtGATA12)
PtrGATA32	Potri.014G058600	PF00320	1497	1397	756	165–200	251	27.96	−0.780	6.42	At2g45050 (AtGATA2)
PtrGATA33	Potri.014G124400	PF00320	1144	942	402	28–63	133	14.7	−0.841	9.77	At5g49300 (AtGATA16)
PtrGATA34	Potri.017G042200	PF06200, PF06203, PF00320	6316	1684	1221	251–288	406	46.03	−0.756	5.36	At3g21175 (AtGATA24)
PtrGATA35	Potri.017G042300	PF00320	356	356	348	61–95	115	12.87	−0.111	9.94	At1g51600 (AtGATA28)
PtrGATA36	Potri.018G044900	PF00320	1864	1342	912	173–206	380	42.11	−0.809	6.22	At5g25830 (AtGATA12)
PtrGATA37	Potri.018G053600	PF00320	1625	1496	1143	173–208	303	33.67	−0.740	8.78	At4g26150 (AtGATA22)
PtrGATA38	Potri.019G033000	PF00320	1808	1460	885	223–258	294	32.20	−0.798	6.18	At4g32890 (AtGATA9)
PtrGATA39	Potri.T158300	PF00320	1516	1308	1065	243–277	354	39.39	−0.835	6.19	At5g25830 (AtGATA12)

AA, amino acid; CDS, coding sequence; GRAVY: grand average of hydropathicity; MM, molecular mass (kDa); p*I*: theoretical isoelectric point.

We assessed the zinc-finger conserved domain of *Populus* and Arabidopsis GATA proteins. The logo pattern from a multiple sequence alignment of 39 *Populus* zinc-finger sequences showed a close similarity with that from 30 Arabidopsis zinc-finger proteins (Supplementary Fig. S3A). Alignment of amino acid sequences for *Populus* and Arabidopsis GATA proteins revealed that zinc fingers of all GATA genes showed high sequence conservation in the α-helix and the four β-foldings (Supplementary Fig. S3B).

To examine phylogenetic relationships among *Populus* GATA family members, we generated an alignment of amino acid sequences for the 39 full-length GATA proteins. The phylogenetic tree of polypeptide sequences is displayed with their domains as well as the exon–intron organization of the corresponding genes (Fig. 1). The *Populus* GATA family was resolved into four subfamilies, with 30 members containing zinc-finger motifs with 18 residues C-X_2_-C-X_18_-C-X_2_-C (subfamilies I, II, and IV), and nine members with 20 residues C-X_2_-C-X_20_-C-X_2_-C (subfamily III). Subfamily I was composed of 18 genes containing two to four exons, of which the last exon encoded an entire zinc-finger motif and carboxy-terminal basic region. All proteins encoded by these genes exhibited a single zinc finger with 18 residues and an acidic amino-terminal region. Subfamily II was composed of 10 genes containing two to four exons, whose members also exhibited 18 residues in a zinc-finger loop, but the zinc-finger motif was located in the last two exons and separated by one intron. Subfamily III included nine genes containing 7–11 exons. All of the corresponding proteins exhibited a single zinc finger with 20 residues. Seven members displayed two additional conserved domains, namely CCT (CONSTANS, CONSTANS -like, and TIMING OF CAB EXPRESSION 1; Robson *et al.*, 2001) and TIFY (Vanholme *et al.* 2007), in the central region of the gene sequence. Subfamily IV comprised two closely related genes that showed a non-homogeneous exon–intron composition and were characterized by one C-X_2_-C-X_18_-C-X_2_-C zinc-finger domain at the N-terminus, and an ASX homology domain (ASXH) at the C-terminus.

To explore potential functions of the *Populus GATA* genes, the promoter regions of *PtrGATA* genes were analysed to identify potential *cis*-elements. Elements responsive to abiotic stress, phytohormone, and developmental processes, especially light-relevant factors, were identified (see Supplementary Fig. S4). The abiotic stress response elements included heat stress (HSE) and low temperature (LTR) responsive elements, MYB binding sites involved in drought inducibility (MBS), a defense and stress response element (TC-rich), and an anaerobic induction element (ARE). Phytohormone response elements, such as the ABA-responsive element (ABRE), ethylene-responsive element (ERE), SA-responsive element (TCA-element), and methyl jasmonate (MeJA)-responsive motifs (CGTCA motif and TGACG motif), were detected. Meristem expression (CAT box and CCGTCC box), zein metabolism regulation (O2 site), and endosperm expression (Skn-1 motif and GCN4 motif) elements were associated with developmental processes. Abundant light-responsive elements, such as BoxI, G-Box, circadian, and Box4, were detected.

### Expression profiles of poplar GATA genes in response to nitrogen

The first plant GATA gene identified, *NTL1*, was isolated as a homolog of the fungus GATA gene *NIT2*, which functions in N metabolism (Daniel-Vedele and Caboche, 1993). Previous studies have reported that plant GATA genes have functions in nitrate metabolism (Bi *et al.*, 2005; Hudson *et al.*, 2011). Given that the most abundant inorganic N source for plants in agricultural soils is nitrate, the majority of previous studies investigating plant N signaling have focused on this compound (Vidal *et al.*, 2015). To determine potential roles of *Populus* GATA genes in response to different N conditions, expression abundance of the 39 homologous *PdGATA* genes was evaluated in the leaf, stem, and root of the rapid-growing *Populus* clone NE-19 treated with three nitrate concentrations for 1 week. The HN (50 mM NO_3_^−^) and LN (0.2 mM NO_3_^−^) treatments changed expression patterns of GATA genes (Fig. 2). In the leaf, 10 *PdGATA* genes were up-regulated under HN (fold change>2, *P*<0.05) and four genes were induced by LN. Four genes (*PdGATA14*, *PdGATA15*, *PdGATA19*, and *PdGATA20*) were induced by both HN and LN. In the root, 10 genes were significantly up-regulated by HN, 16 genes were induced by LN, and eight genes were induced by both HN and LN. In stem tissues, eight *PdGATA* genes were up-regulated by HN, five genes were induced by LN, and three genes were induced by both HN and LN. The majority of paralogous gene pairs showed distinct expression patterns, whereas a small number showed an almost identical expression pattern in different tissues, such as *PdGATA4* and *PdGATA17*.

### CRISPR/Cas9-mediated mutagenesis of poplar *GATA19*/*GNC*

**Fig. 3.**
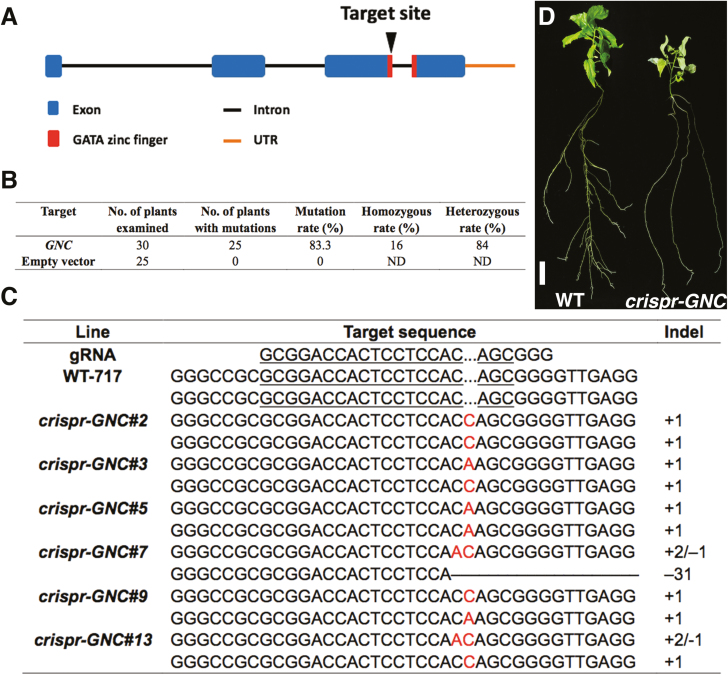
CRISPR/Cas9-mediated mutagenesis of *Populus GNC* gene. (A) The selected target site in the *Populus GNC* locus. (B) Determination of mutation events in transgenic *Populus* plants. ND, not determined. (C) CRISPR/Cas9-induced mutagenesis at target sites of *Populus GNC* gene sequences. (D) Phenotypic comparison of the *crispr-GNC* mutant and wild-type *Populus* plants. Scale bar=1 cm.

**Fig. 4.**
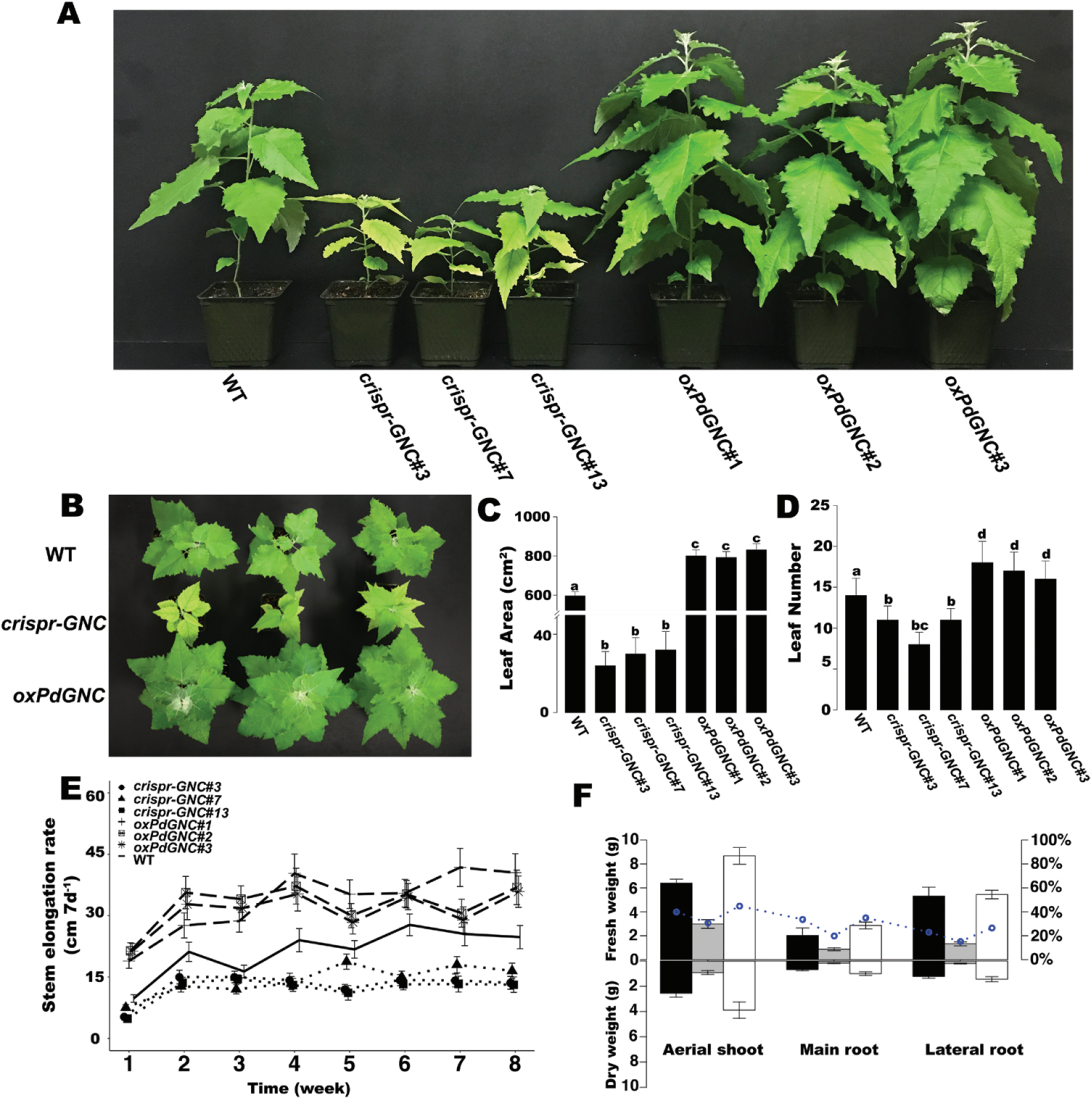
Phenotypic effects of *PdGNC* on growth and development of 8-week-old wild-type (WT), *crispr-GNC* mutant, and *PdGNC-*overexpression (*oxPdGNC*) plants. (A, E) Height. (B, C) Leaf area. (D) Leaf number. (F) Biomass accumulation. The parameters fresh weight (FW) and dry weight (DW) were compared among WT, *crispr-GNC*, and *oxPdGNC* plants. Circles represent the DW/FW values. The data are the mean ±SD (*n*=10). Bars with different letters indicate a significant difference (*P*<0.05; one-way ANOVA).

Expression profiles revealed that the *Populus* GATA gene *GATA19*/*GNC* was strongly up-regulated in *Populus* leaves in response to nitrate treatment. Analysis of phylogenetic relationships and multiple sequence alignment revealed that *Populus* GATA19 was phylogenetically closer to, and showed higher sequence similarity with, AtGNC (AT5G56860). The *AtGNC* mutant *gnc* exhibits yellow leaves. Our previous research showed that the Arabidopsis complementary lines *gnc*/*PdGNC* were restored to the WT chlorophyll levels (An *et al.*, 2014), suggesting that *Populus* GATA19 and AtGNC were orthologous (see Supplementary Fig. S3B). AtGNC has been reported to participate in the correlation between N and C metabolism in Arabidopsis (Bi *et al.*, 2005; An *et al.*, 2014), but functional interpretation is not exhaustive especially in woody plants. The CRISPR/Cas9 system was employed to edit the *Populus* genomic sequence and disrupt *GNC* expression. The potential target site was located at the beginning of the coding sequence of the GATA zinc-finger domain so as to break the indispensable functional structure (Fig. 3A). Notably, nucleotide sequences of the GATA zinc-finger domain in all 39 genes were not identical and matched (Supplementary Fig. S5). The sequencing results also indicated that no off-target events happened within these sequences. With *Agrobacterium*-mediated transformation of the CRISPR/Cas9 module into *Populus* clone 717, 25 of 30 examined transgenic plants were successfully mutated (Fig. 3B) by editing the target site by insertion or deletion of a small number of nucleotides (Fig. 3C; Supplementary Fig. S6). The CRISPR/Cas9 system created mutation events in both of two *Populus GNC* allelic sequences, and several biallelic mutants were generated from different editing events. Nucleotide insertions/deletions led to a frameshift mutation and change in the translated amino acids, thereby disrupting expression of the *Populus* GNC protein. Loss-of-function *crispr-GNC* mutant plants showed visibly pale green leaves with significantly decreased chlorophyll content (Fig. 3D). In addition, *crispr-GNC* plants showed retarded growth compared with that of the controls (Fig. 4).

### Expression modification of *PdGNC* reveals *GNC*-associated regulation of vegetative growth, chlorophyll content, and vascular development

**Fig. 5.**
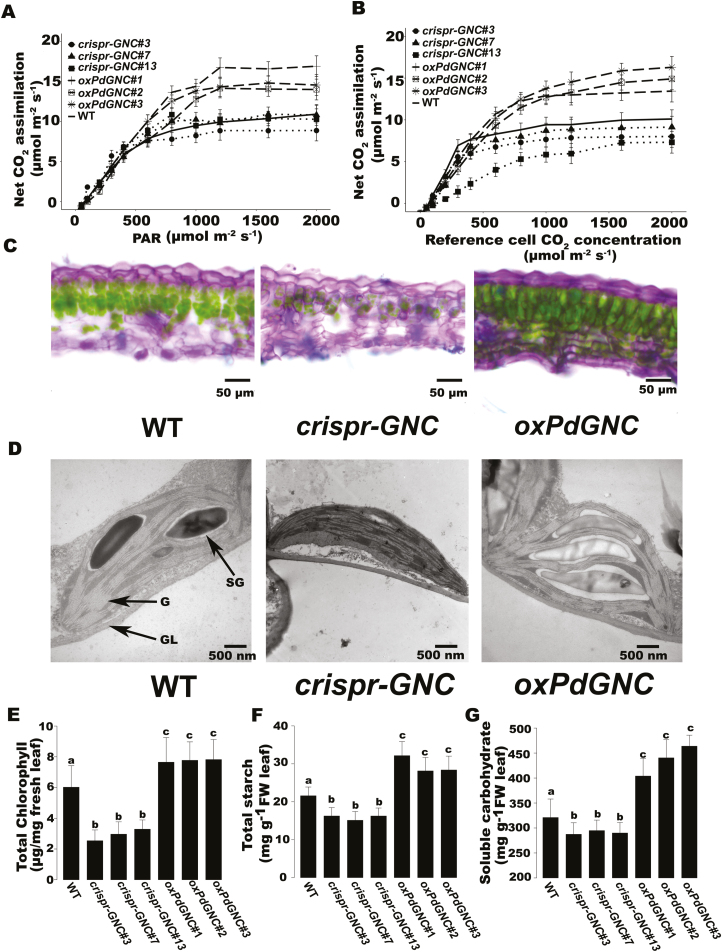
Photosynthesis and chlorophyll content modulated by *PdGNC*. (A) *A*–*C*_i_ curve. (B) *A*–light response curve. (C) Transverse sections of the fifth functional leaf. Scale bar=50 μm. (D) Chloroplast ultrastructure. Scale bar=500 nm. G, grana; GL, grana lamellae; SG, starch granules. The data are the mean ±SD (*n*=10). Bars with different letters indicate a significant difference (*P*<0.05; one-way ANOVA). (E) Total chlorophyll content of leaves. (F) Total starch content of leaves. (G) Soluble carbohydrate content of leaves.

To further investigate the role of *Populus GNC* in plant growth, we cloned *PdGATA19*/*PdGNC* from *Populus* clone NE-19 and transformed the gene into *Populus* clone 717. Transgenic overexpression plants (*oxPdGNC*) exhibited darker green leaves and faster growth than the control WT under the normal condition. Analysis of relative growth rates measured at weekly intervals revealed that *oxPdGNC* plants showed a faster growth rate than the WT and *crispr-GNC* mutant (Fig. 4A, E). The *oxPdGNC* plants also showed increased leaf area (Fig. 4B, C) and leaf number (Fig. 4B, D), as well as higher photosynthetic rates, which were 20–28% and 100–110% higher than those of the WT and *crispr-GNC* mutant, respectively. Photosynthesis–light and photosynthesis–CO_2_ response curves indicated that the *oxPdGNC* plants showed higher photosynthetic capability than the WT and *crispr-GNC* mutant (Fig. 5A, B). Photosynthetic rates per unit leaf volume of *oxPdGNC* plants were 20–28% and 30–60% higher than those of the WT and *crispr-GNC* mutant, respectively (see Supplementary Fig. S7). Higher photosynthetic efficiency contributed to greater biomass accumulation. The fresh weight (FW) of aboveground biomass of overexpression lines increased by ~35.5% compared with the WT and ~190% compared with the *crispr-GNC* mutant; the dry weight (DW) increased by ~51% compared with that of the WT and ~320% compared with the *crispr-GNC* mutant. The DW/FW values of the above- and belowground biomass of overexpression lines were all larger than those of the WT and *crispr-GNC* lines. The *crispr-GNC* mutant showed the slowest growth rate and lowest biomass accumulation (Fig. 4F).

Coincident with higher photosynthesis rates, *oxPdGNC* plants displayed higher chlorophyll content. The total chlorophyll content of *oxPdGNC* plants was 25–30% and 105–130% higher than those of the WT and *crispr-GNC* mutant, respectively (Fig. 5C, E). Transverse sections of 2-month-old fully expanded leaves showed the absence of palisade parenchyma and spongy mesophyll in the *crispr-GNC* mutant. In contrast, chloroplast biogenesis in the leaf mesophyll was enhanced by *PdGNC* overexpression (Fig. 5E). Further analysis of cellular organelles by TEM indicated that in the WT chloroplasts were distributed uniformly and thylakoids were tightly stacked. In contrast, in *crispr-GNC* plants, the thylakoids were slightly scattered and very few or no visible starch granules were observed (Fig. 5D). The number of thylakoids in *oxPdGNC* and the WT differed slightly, but the grana number in *oxPdGNC* was 2.2-fold higher than that in *crispr-GNC* plants. The total starch content of overexpression lines increased ~36.9% compared with the WT and ~68.5% compared with *crispr-GNC* plants (Fig. 5F). Soluble carbohydrate content increased ~35.8% compared with the WT and ~55.4% compared with the *crispr-GNC* mutant (Fig. 5G).

**Fig. 6.**
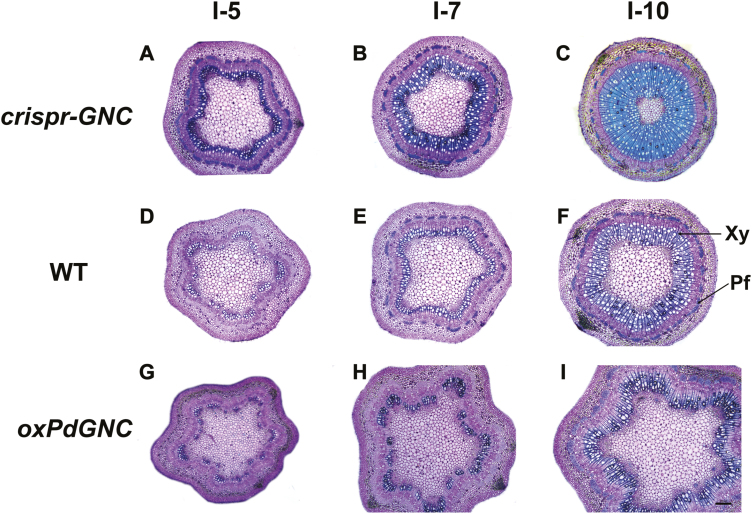
Transverse sections of stem from 2-month-old *crispr-GNC* mutant, wild type (WT), and *PdGNC-*overexpression (*oxPdGNC*) plants. (A, D, G) Magnified view of the fifth internode of *crispr-GNC*, WT, and *oxPdGNC* stem during primary growth. (B, E, H) Magnified view of the seventh internode of *crispr-GNC*, WT, and *oxPdGNC* stem during the transition to secondary growth. (C, F, I) Magnified view of the 10th internode of *crispr-GNC*, WT, and *oxPdGNC* stem. Pf, phloem fiber; Xy, xylem. Scale bar=100 μm. Each experiment comprised three biological replicates.

The dwarf phenotype of the *crispr-GNC* mutant encouraged us to explore variation in stem structure. Transverse sections of the stem from the shoot apex to the base showed a gradual transition from primary to secondary growth in the stem, and that stems of *crispr-GNC* plants produced a higher number of secondary xylem cells than WT and *oxPdGNC* plants at the same developmental stage. The *crispr-GNC* mutant formed a mature xylem ring around the cambium at the fifth internode (I-5), whereas at the same internode WT and *oxPdGNC* plants produced very few secondary growth cells. At the seventh internode (I-7), *crispr-GNC* plants had produced 2- or 3-fold more secondary xylem cell layers compared with I-5, whereas WT plants had just formed the xylem ring and in *oxPdGNC* plants only a small number of mature xylem cells had differentiated. At the tenth node (I-10), *oxPdGNC* plants had just begun to develop the lignified xylem ring (Fig. 6).

### Transcriptional model modification by poplar GNC overexpression or suppression

**Fig. 7.**
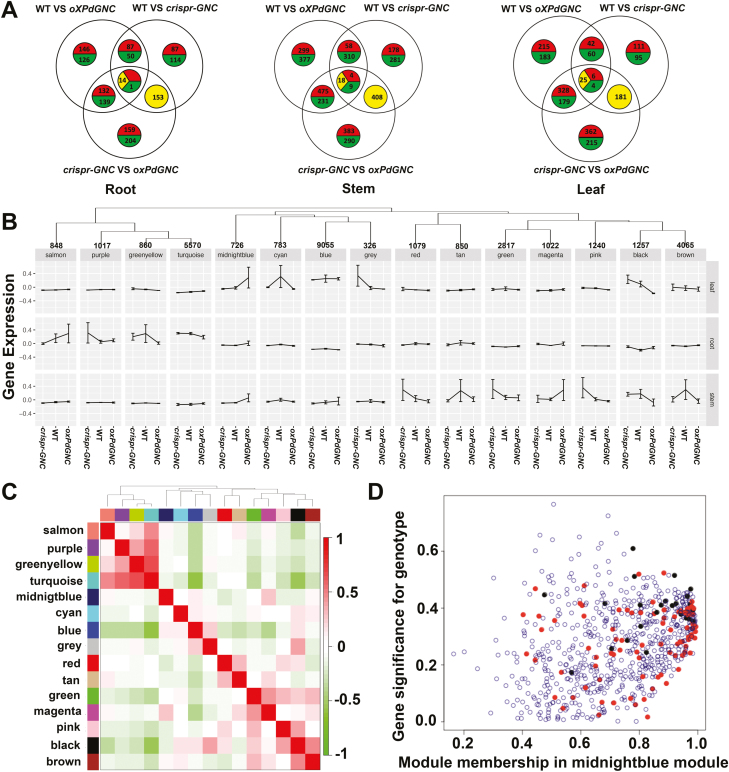
Gene differential expression and co-expression network of *Populus GNC* modified plants. (A) Gene differential expression in the root, stem, and leaf of *crispr-GNC*, wild type, and *PdGNC-*overexpression (*oxPdGNC*) plants. The red part represents the genes up-regulated in all comparisons, the green represents the genes down-regulated in all comparisons, and the yellow part represents the genes down-regulated or up-regulated in different comparisons. (B) Eigengene expression profiles of modules. (C) Correlation between modules. Each row and column corresponds to a module eigengene. (D) Scatterplot of gene significance for genotype versus module membership of all genes in the module midnightblue. Red points are transcription factor genes, black points are ethylene response-related transcription factors.

**Fig. 8.**
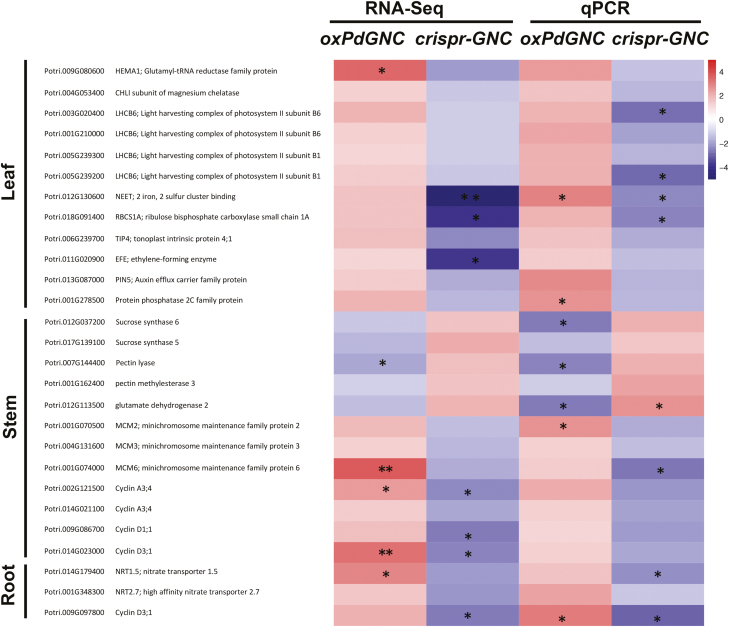
Heatmap of expression profiles of differentially expressed genes between *PdGNC-*overexpression (*oxPdGNC*), *crispr-GNC*, and wild type plants. qPCR was used to analyse expression levels of genes differentially expressed in the leaf, stem, and root. Asterisks indicate a significant difference (*P<0.05, **P<0.01; one-way ANOVA).

The phenotypic variation motivated us to further explore transcriptional changes in the leaf, stem, and root induced by *Populus GNC* overexpression and suppression. Differential gene expression analysis showed that modification of the *GNC* transcript level resulted in different transcriptional reprogramming between genotypes and tissues (Fig. 7A). In leaves, the hormone response, such as ethylene, auxin, ABA, and jasmonic acid, clustered around many up-regulated genes induced by *PdGNC* overexpression (see Supplementary Dataset S1). The ethylene synthesis gene *Ethylene-Forming Enzyme* (*EFE*) and the auxin efflux carrier gene *PIN5* were up-regulated in *oxPdGNC* plants and down-regulated in *crispr-GNC* lines. The nitrogen transporter gene *Tonoplast Intrinsic Protein 4* (*TIP4*) exhibited the same expression profile (Fig. 8). In stems, several genes involved in cell wall biogenesis and carbohydrate metabolism were up-regulated in overexpression lines. Many genes associated with the cell cycle and cell division were down-regulated in *crispr-GNC* plants. In roots, many genes associated with cell wall biosynthesis, the cell cycle, and nitrate transport were up-regulated in overexpression plants (Supplementary Dataset S1).

Additionally, WGCNA was employed to define different co-expressed modules of genes that were expressed across different tissues of WT, *PdGNC* overexpression, and CRISPR-induced plants. A total of 15 modules were determined by the gene co-expression relationship (Fig. 7C). The plot of eigengene values in each module revealed the tissue-specific variation in gene expression in different *PdGNC*-modified plants and in different gene co-expression modules (Fig. 7B). All modules were also annotated with GO enrichment terms to shed additional light on functional properties of the modules.

Eigengene expression in module midnightblue was up-regulated in all tissues, and GO annotation showed that the module was enriched in the regulatory processes responses to hormones and nitrogen metabolism. Sixty transcription factors, including members of the ERF, WRKY, C2H2, GRAS, MYB, and NAC transcription factor families, were differentially expressed in this module (Fig. 7D; Supplementary Dataset S2). Twenty ERF and five MYB genes were up-regulated in different tissues of *oxPdGNC* lines in the ethylene response process. For the ABA response process, except transcription factors of the ERF, C2H2, and MYB, *Protein Phosphatase 2C* (*PP2C*), and *Plasma membrane Intrinsic Protein 3* (*PIP3*) were up-regulated in the leaves. With regard to N metabolism processes, *Asparaginase B1* (*ASPGB1*) and *Phenylalanine Ammonia Lyase 1* (*PAL1*) were induced in the leaves. In addition, two glutamyl-tRNA reductase (*HEMA1*) genes were up-regulated in the leaves. The latter protein functions as a crucial rate-limiting enzyme in the early steps of chlorophyll biosynthesis.

The module blue showed significant tissue-specific expression profiling and genes within this module were mainly expressed in the leaves. GO annotation revealed enrichment in a variety of biological processes and molecular structures involved in photosynthesis, such as chloroplast organization, photosynthetic electron transport chain, and chlorophyll binding. Differentially expressed genes within this module mostly participated in the chloroplast envelope and stroma, and ion homeostasis. Two light harvesting complex B1 subunit protein (*LHCB1*) genes and two B6 subunit (*LHCB6*) genes were up-regulated in *oxPdGNC* and down-regulated in *crispr-GNC* plants. These proteins are components of PSII antenna proteins and responsible for light harvesting and electron transport rate limitation (de Bianchi *et al.*, 2008). In addition, two Mg chelatase subunit I (*CHLI*) genes and the subunit H gene *CHLH*, and a *NEET* gene functioning as a Fe–S cluster donor for ferredoxins primarily in the photosynthetic electron transport chain also displayed parallel expression patterns (Nechushtai *et al.*, 2012). The biological processes that were enriched in these modules demonstrated the important roles of *Populus GNC* genes in photosynthesis.

## Discussion

### Evolution and divergence of genes encoding GATA transcription factors

Transcription factors regulate expression of genes that mediate diverse biological processes in cells and organisms, and are employed as a principal source of the diversity and change that underlie evolution (Riechmann and Ratcliffe, 2000). Investigation of the *Populus* genome revealed 39 *Populus* GATA transcription factor genes, which indicated that the woody tree *Populus* has a higher number of GATA family members than the 30 and 28 *GATA* genes identified in Arabidopsis (*AtGATA*s) and rice (*OsGATA*s), respectively (Reyes *et al.*, 2004; Bi *et al.*, 2005). Variation in GATA family gene number may be attributed to gene or genome duplication, which has been considered to be a primary source of genetic novelty and progress in plant evolution (Yang *et al.*, 2006). Previous studies revealed that *GATA* genes can be classified into seven subfamilies (I–VII) based on their phylogenetic relationships (Reyes *et al.*, 2004). *Populus* and Arabidopsis lack the rice-specific subfamilies V, VI, and VII (see Supplementary Table S4). Concerning the GATA subfamily classification in *Populus*, Arabidopsis, and rice, we inferred that both woody and herbaceous eudicotyledons harbor GATA subfamilies I, II, III, and IV, but subfamily IV is absent in monocotyledons. This result further verifies the hypothesis that subfamilies I, II, and III arose before divergence of monocotyledons and eudicotyledons, whereas subfamilies IV, V, VI, and VII may have been lost or arose after divergence of monocotyledons and eudicotyledons (Reyes *et al.*, 2004). Evolutionary variation in monocotyledon and eudicotyledon plants may have contributed to functional divergence of *GATA* genes in subfamilies IV, V, VI, and VII, but currently little information is available on functional differences among these subfamilies.

### Functional variance of GATA transcription factors in poplar

Mapping the reported functions of characterized *GATA* genes to the phylogenetic tree revealed that similar gene functions were clustered in the same subfamily. The Arabidopsis homologous genes in subfamily I are mainly involved in light signaling pathways (Manfield *et al.*, 2007; Luo *et al.*, 2010). Although *Populus* genes in subfamily I have not been reported until now, *cis*-element analysis of the promoter region of *Populus* genes revealed abundant elements associated with light responses, implying that these genes have potential roles in light responses.

Subfamily II contains many genes that function in flowering regulation. Recent studies reveal that subfamily II could be subdivided into two classes respectively characterized by an N-terminal HAN (HANABA TARANU) and a C-terminal LLM (leucine–leucine–methionine) domain. GATA genes that contain the HAN domain play roles in floral development (Zhang *et al.*, 2013), whereas genes that contain the LLM domain, such as *GNC* and *CGA1*/*GNL* (Richter *et al.*, 2013; Behringer and Schwechheimer, 2015; Ranftl *et al.*, 2016), function in the control of flowering time, as well as chloroplast development and vegetative growth. These studies show that different gene structures combined with the GATA core domain may contribute to neofunctionalization of *GATA* genes.

Atypical architectures of the CCT and TIFY domains identified in the *Populus* GATA subfamily III members (Fig. 1) would develop additional functions, such as stress and phytohormone response (Shikata *et al.*, 2004; Vanholme *et al.*, 2007; Ye *et al.*, 2009; Chung *et al.*, 2009; Kazan and Manners, 2012; Pérez *et al.*, 2014). The CCT domain is mainly present in subfamily III in both *Populus* and Arabidopsis, which is suggestive of conserved functions for this subfamily in woody and herbaceous plants. Members of the subfamily are an important regulator and a central component of the photoperiodic floral progress and circadian regulation (Hayama and Coupland, 2003). Although several homologs of the genes that govern flowering in Arabidopsis are present in trees (Böhlenius *et al.*, 2006; Mohamed *et al.*, 2010; Hsu *et al*., 2011), it will be necessary to investigate the tree genes in tree-specific processes. The GATA-CCT-dependent pathway would provide insights to elucidate such tree-specific processes for flowering and circadian regulation. Accompanied by the CCT domain, the TIFY domain is also present in subfamily III. The TIFY domain was first identified in Arabidopsis *AtGATA25* (At4g24470), which contains the CCT domain in addition to TIFY (Nishii *et al.*, 2000). Several screenings have revealed a link between TIFY proteins and the jasmonic acid-related (JA) response (Ye *et al.*, 2009). Three Arabidopsis GATA subfamily III genes, namely *AtGATA24*/*ZML1* (At3g21175), *AtGATA25*/*ZIM* (At4g24470), and *AtGATA28*/*ZML2* (At1g51600), contain CCT and TIFY domains and are involved in JA responses (Vanholme *et al.*, 2007; Kazan and Manners, 2012; Pérez *et al.*, 2014). The *Populus* GATA subfamily III genes *PtrGATA5*, *PtrGATA30*, and *PtrGATA34*, which are homologs of *AtGATA24*, *AtGATA25*, and *AtGATA28*, respectively, have the JA-related CGTCA motif and TGACG motif in the promoter sequences, in addition to the CCT and TIFY domains, which implies that these GATA genes may also participate in the JA response. Coupled with the presence of stress-related elements, we suggest that *Populus* GATA genes have potential roles in response to additional heat/low temperature, drought, and other external signals. They may contribute to the evolution of a greater number of traits adaptive to environmental stress conditions for perennial woody plants, compared with annual herbaceous plants.

### Conserved roles of poplar GATA transcription factors in chlorophyll biosynthesis and photosynthesis

The phenotypic traits associated with *GNC* in Arabidopsis (Hudson *et al.*, 2011) and *Populus* indicate that plant *GNC* genes show conserved functions in chlorophyll biosynthesis and starch accumulation in both herbaceous and woody plants (Fig. 3D; Fig. 5). The chlorophyll content reflects the N status and increase in chlorophyll content provides an improved capacity to convert light energy to chemical energy and enhanced carbohydrate accumulation (Dordas and Sioulas, 2008). Chlorophyll is a type of tetrapyrrole and the *PdGNC*-induced *HEMA* gene encodes an enzyme that participates in biosynthesis of 5-aminolevulinic acid, which is a universal precursor of tetrapyrrole synthesis (Jahn *et al.*, 1992). This important rate-limiting step, which is boosted by *PdGNC* overexpression, would expedite chlorophyll biosynthesis and increase chlorophyll content. In addition, up-regulation of Mg-chelatase genes further stimulates photosynthetic pigment biosynthesis. The other integral LHCB protein genes induced by *PdGNC* also contribute to the robustness of the PSII light harvesting complex, which increases the capability and efficiency of light absorption (de Bianchi *et al.*, 2008). The harvested energy is transferred by the photosynthetic electron transfer chain, which is promoted by *PdGNC*-regulated iron–sulfur cluster metabolism. Proteins containing iron–sulfur clusters include ferredoxins and NADH dehydrogenase, which participate in the oxidation–reduction reactions in photosynthesis. The elevated chlorophyll content and photosynthetic efficiency induced by *PdGNC* overexpression will lead to production of a larger C source for plant growth, resulting in accelerated growth and enhanced biomass accumulation.

### Divergent roles of poplar GATA transcription factor in plant growth

In contrast to the notable differences in growth and morphology of *oxPdGNC* plants and the *crispr-GNC* mutant (Fig. 4), neither the Arabidopsis *GNC* (*AtGNC*) overexpressing transformant nor the *gnc* mutant show notable phenotypic variation with regard to overall growth (Hudson *et al.*, 2011). The difference in impacts of *GNC* gene modification between Arabidopsis and *Populus* may be attributable to the divergent functions of *GNC* genes in different growth regulatory systems between herbaceous and woody plants. *AtGNC* does not have significant effects on plant growth, except for chlorophyll content and starch production. Arabidopsis has a short life cycle, and vegetative growth strongly impacts on reproduction. Greater material reserves and energy accumulation are utilized for reproductive growth, and proportionally less for vegetative growth. However, the perennial woody tree *Populus* has a longer life cycle and requires substantially greater quantities of nutrients and energy for vegetative growth. Thus, *PdGNC*, the counterpart of *AtGNC* in a woody tree system, is involved in not only chlorophyll biosynthesis and starch accumulation, but also promotion of plant growth and maintenance of overall plant architecture. Transcriptomic data indicated that a large number of cyclin genes (*CYCAs*, *CYCBs*, and *CYCDs*) and mini-chromosome maintenance protein genes (*MCMs*) were down-regulated in the *crispr-GNC* lines (Fig. 8). Decreased expression levels of these important regulators of DNA replication and the cell cycle may be responsible for the retarded growth of *crispr-GNC* plants (Tuteja *et al.*, 2011). In stems of the *crispr-GNC* plants, pectin methylesterase genes (*PMEs*) were up-regulated. The proteins encoded by these genes demethylesterify homogalacturonan, which is the main component of pectic polysaccharides in the cell wall. These low-methylesterified homogalacturonans are degraded by pectin lyases and polygalacturonases to form oligogalacturonides, which can loosen the cell wall and eventually lead to vertical growth inhibition (Ridley *et al.*, 2001). In addition, sucrose synthase genes (*SUSs*) were up-regulated. These genes control C flow during cell wall biosynthesis (Fujii *et al.*, 2010). Higher quantities of carbohydrates are incorporated in the secondary cell wall of woody cells but are not necessarily assigned to other normal plant vegetative growth processes, such as increment in shoot height and leaf area. The genes associated with carbohydrate transport and the electron transport needed for sugar transport were down-regulated in the *crispr-GNC* mutant. Therefore, we infer that *Populus GNC* genes participate in the cell cycle and C distribution. Thus, *PdGNC* positively regulates C fixation and distributes C metabolites for plant vegetative growth to attain greater biomass accumulation.

The study of *Populus GATA* genes enables clarification of the functional conservation and divergence of *GATA* genes in herbaceous and woody plants. Although the majority of *Populus GATA* genes are of unknown function, the present comparative phylogenetic and expression analysis provides a basis for future functional studies of the GATA transcription factor family in *Populus* and other woody plants. In particular, the responses of *GNC* genes to N stress will be a focus of future research.

## Supplementary data

Supplementary data are available at *JXB* online.

Dataset S1. Gene ontology (GO) annotation and enrichment analysis of differentially expressed genes in the leaf, stem, and root of *oxPdGNC*, wild type, and *crispr-GNC* poplar plants.

Dataset S2. Summary of transcription factors differentially expressed in module midnightblue.

Fig. S1. *PdGNC* gene expression levels in different overexpression lines.

Fig. S2. Location of GATA gene family members on *Populus* chromosomes.

Fig. S3. Conserved core sequence logos and amino acid sequence alignment of *Populus* and Arabidopsis GATA zinc-finger motifs.

Fig. S4. Overview of predicted *cis*-acting elements in the promoter region of *PtrGATA* genes.

Fig. S5. Alignment of nucleotide sequences of the GATA zinc-finger domain in all 39 *Populus* GATA genes.

Fig. S6. Representative Sanger sequencing chromatograms at the target site.

Fig. S7. Comparison of net photosynthetic rate per unit leaf volume.

Table S1. The qPCR primers used for expression profiles of *Populus GATA* genes in response to nitrogen.

Table S2. Primers used for qPCR analysis of genes differentially expressed in the leaf, stem, and root of *oxPdGNC*, *crispr-GNC*, and wild-type *Populus* plants.

Table S3. Primers used in CRISPR/Cas9-mediated mutagenesis of *Populus GNC* gene.

Table S4. Summary of GATA subfamilies in *Populus*, Arabidopsis, and rice.

erz564_suppl_Supplementary_Figure_S1_S7

erz564_suppl_Supplementary_Tables_S1_S4

erz564_suppl_Supplementary_Dataset_S1

erz564_suppl_Supplementary_Dataset_S2
