# Supplementary material for: The GATA transcription factor GNC plays an important role in photosynthesis and growth in poplar
Source: J Exp Bot. 2019 Dec 24;71(6):1969–84. doi: 10.1093/jxb/erz564 (PMC7094078; doi:10.1093/jxb/erz564)
Supplement: erz564_suppl_Supplementary_Figure_S1_S7 [file erz564_suppl_supplementary_figure_s1_s7.pdf]

Supplementary Figure 1

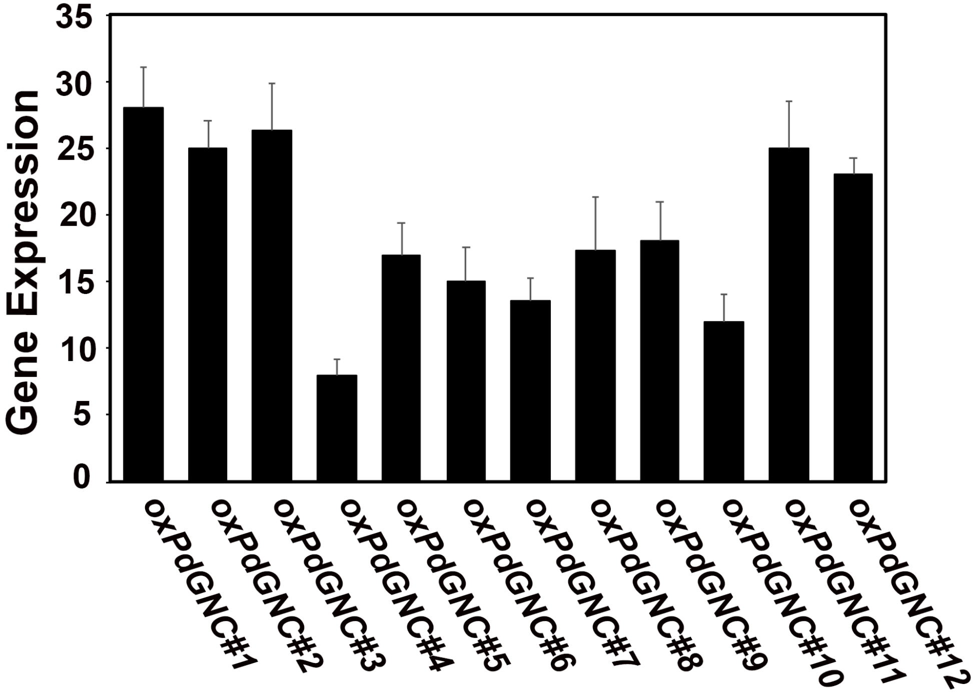

Fig. S1. *PdGNC* gene expression levels in different overexpression lines.

Supplementary Figure 2

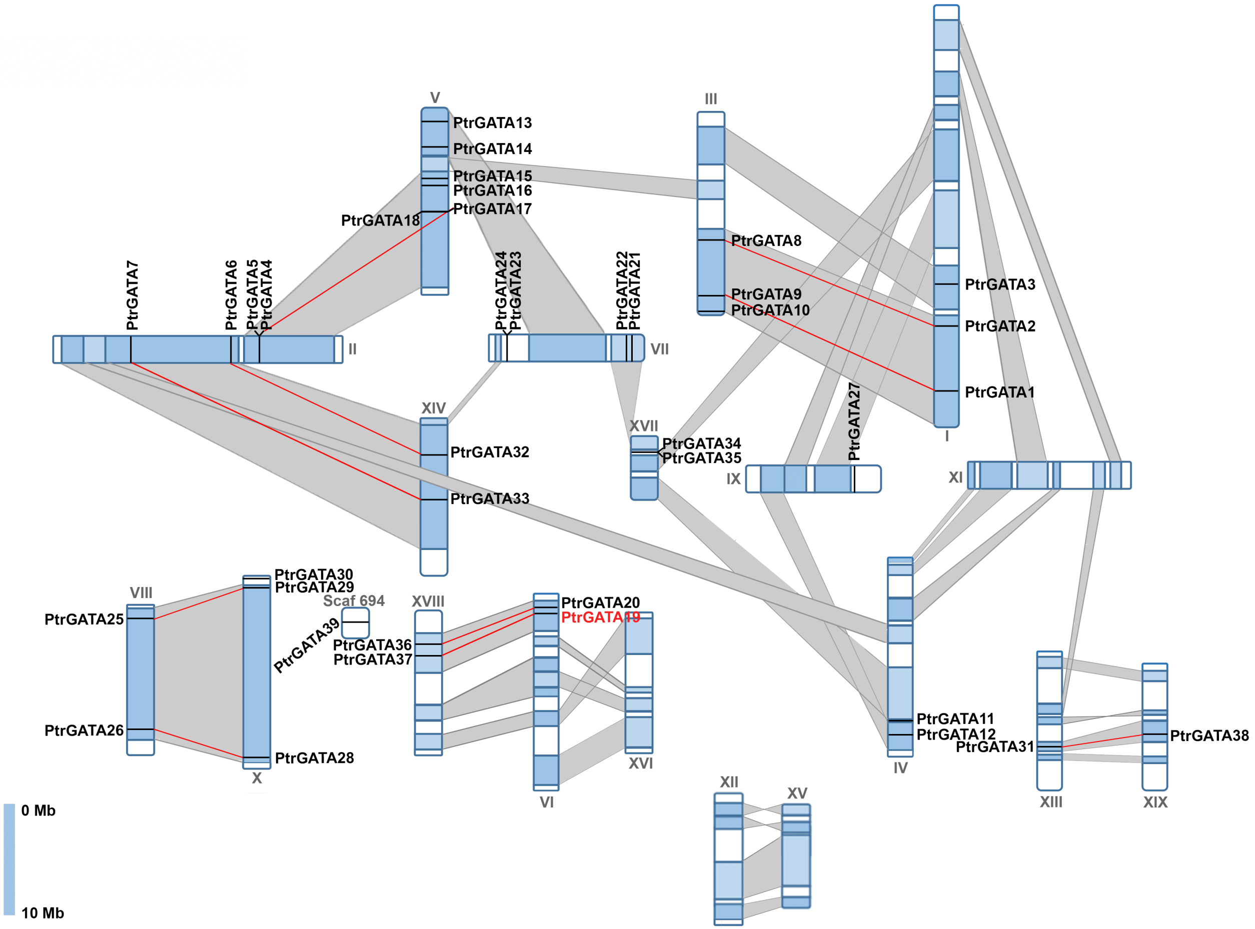

Fig. S2. Location of GATA gene family members on *Populus* chromosomes. The schematic overview of *Populus* chromosome reorganization after the most recent genome-wide salicoid-specific duplication event is adapted from Tuskan *et al*. (2006). The 19 *Populus* chromosomes are shown with the linkage group numbers and size. The scale is one megabase (Mb). Lines connect paralogous gene pairs. Red lines represent the segmental duplication.

Supplementary Figure 3

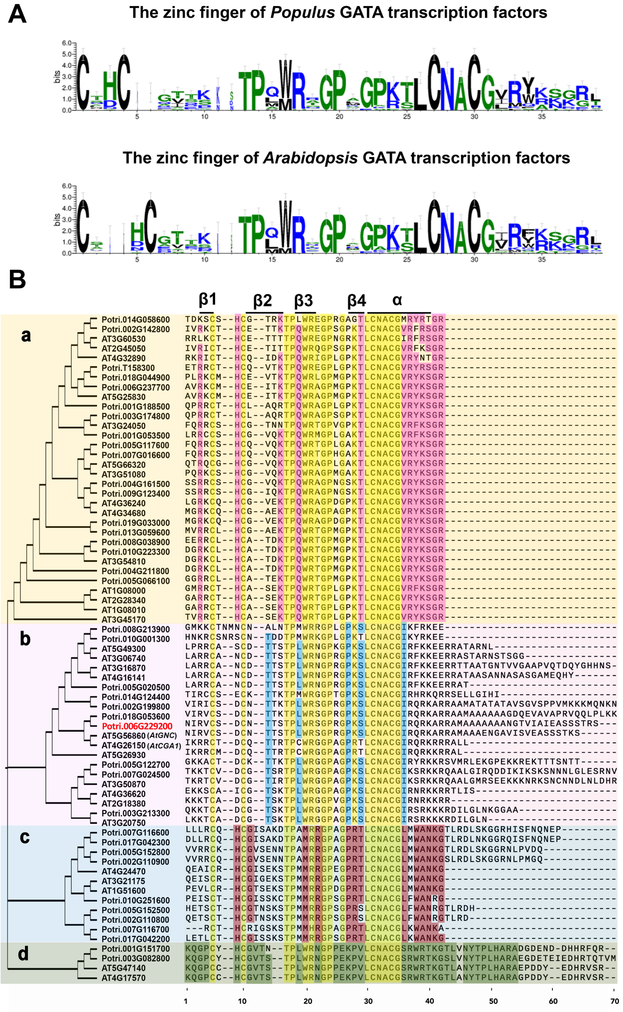

Fig. S3. Conserved core sequence logos and amino acid sequence alignment of *Populus* and Arabidopsis GATA zinc-finger motifs. (A) Overall height of each stack indicates the sequence conservation at that point. The height of each letter (representing a residue) shows the relative frequency of the corresponding residue at that position. (B) Four clusters (a, b, c, and d) are categorized according to analysis of phylogenetic relationships and the residue loop and protein characters. Residues specifically conserved in four clusters are highlighted in different colors. Several amino acids in the zinc finger are highly conserved, such as Cysteine (5, 10), Threonine (17), Proline (18, 24), and Glycine (23). The second pairs of Cysteine residues (LCNACG) are conserved in all sequences. The top shows secondary structure elements of zinc-finger motifs. The gene Potri.006G229200 (*PtrGATA19*) is highlighted in red.

Supplementary Figure 4

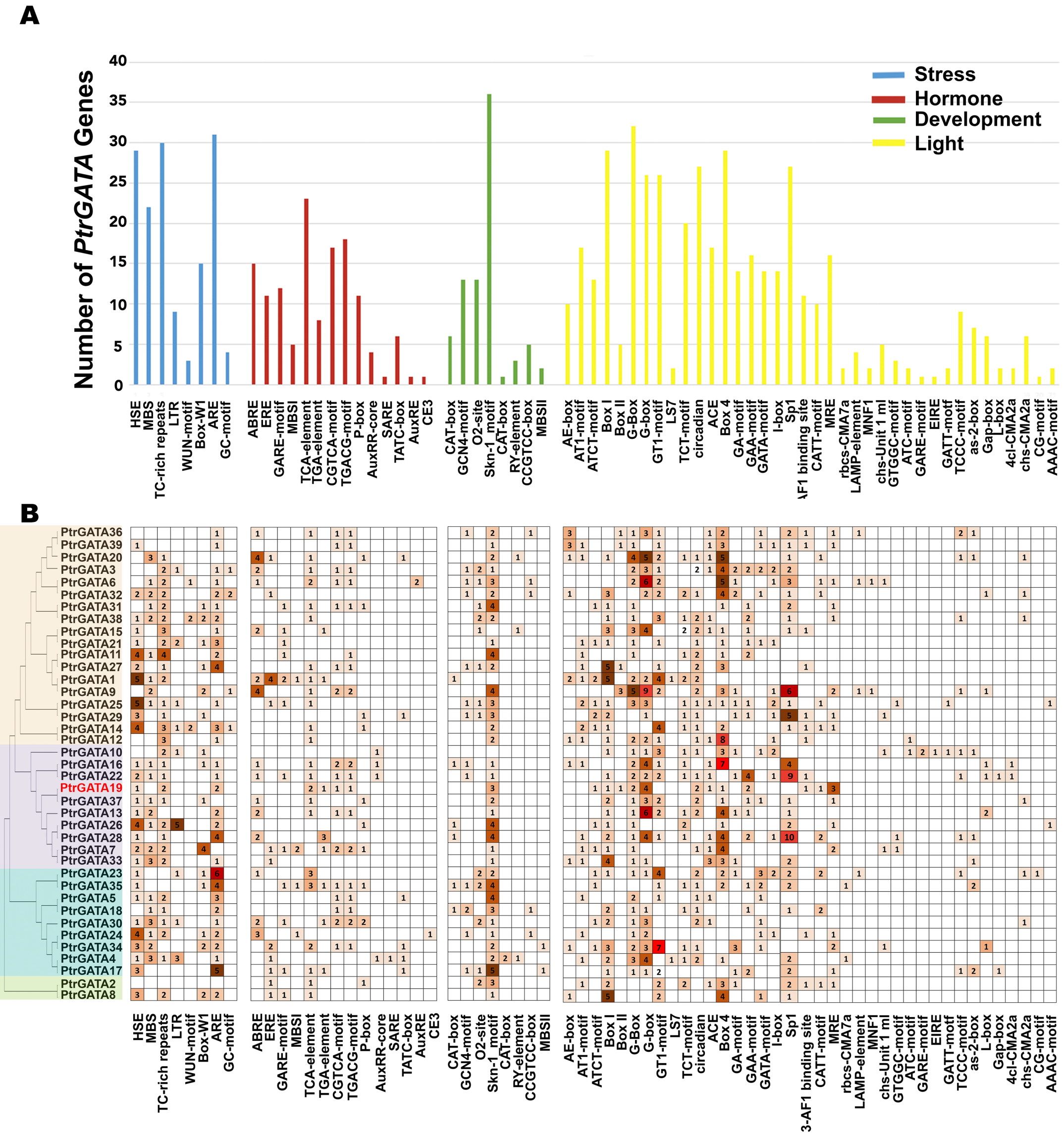

Fig. S4. Overview of predicted *cis*-acting elements in the promoter region of *PtrGATA* genes. (A) Number of *PtrGATA* genes containing the corresponding *cis*-acting elements. (B) Number of occurrences of each *cis*-acting element in the promoter region of each *PtrGATA* gene.

Supplementary Figure 5

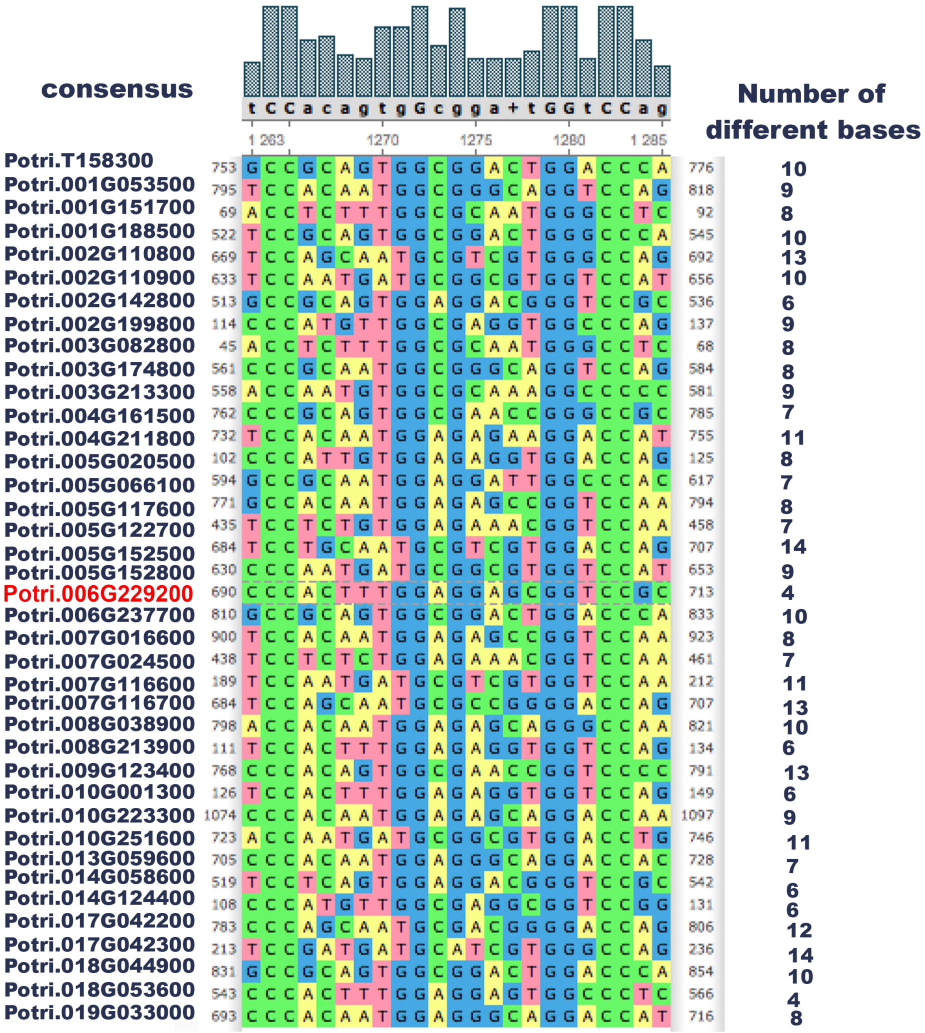

Fig. S5. Alignment of nucleotide sequences of the GATA zinc-finger domain in all 39 *Populus* GATA genes.

Supplementary Figure 6

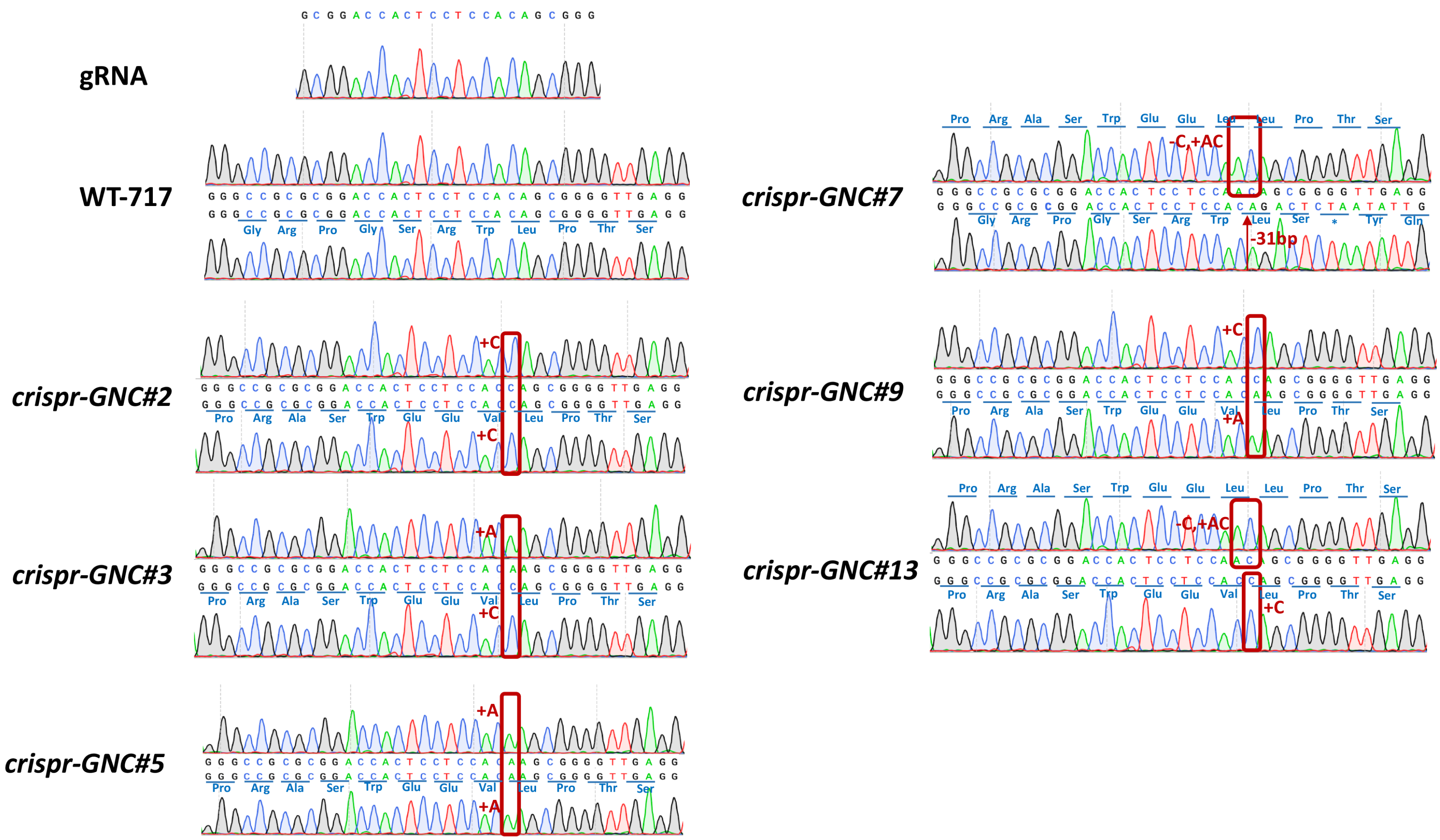

Fig. S6. Representative sanger sequencing chromatograms at the target site.

Supplementary Figure 7

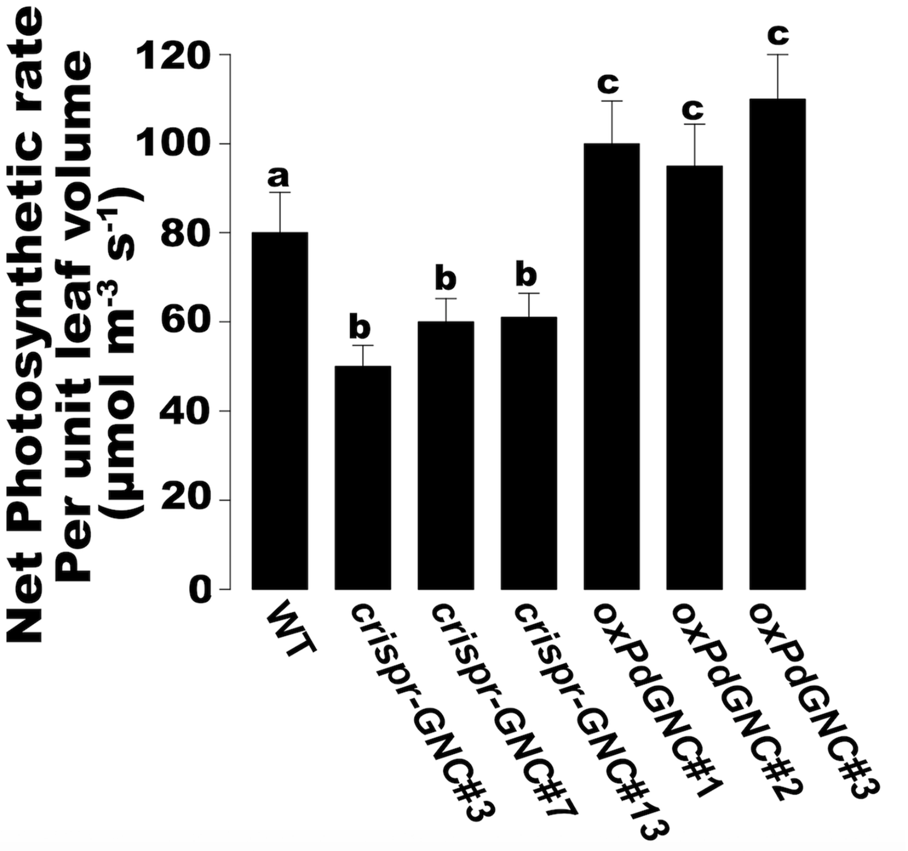

Fig. S7. Comparison of net photosynthetic rate per unit leaf volume.
