# Supplementary material for: The GATA transcription factor GNC plays an important role in photosynthesis and growth in poplar
Source: J Exp Bot. 2019 Dec 24;71(6):1969–84. doi: 10.1093/jxb/erz564 (PMC7094078; doi:10.1093/jxb/erz564)
Supplement: erz564_suppl_Supplementary_Tables_S1_S4 [file erz564_suppl_supplementary_tables_s1_s4.pdf]

Table S1 The qPCR primers used for expression profiles of *Populus GATA* genes in response to nitrogen

Name	Gene model ID	Primer-F	Primer-R
PtrGATA1	Potri.001G053500	GCGTTCCCAACAGTCGAAAC	GCTACGAGCCTTAACAGGCA
PtrGATA2	Potri.001G151700	TGCAATGCATGTGGATCACG	GAGCAACACCTTCAGCAACC
PtrGATA3	Potri.001G188500	GGAGAGCCCAGATCCAAACC	CCCACATGCATTACACAGCG
PtrGATA4	Potri.002G110800	ACAGAGAATGCACCGCAAGA	TCTGAAAGAGGGGTGCCATC
PtrGATA5	Potri.002G110900	AACCTCAACGAGCAGCTTCA	GTGCTGAATCCTGACCACCA
PtrGATA6	Potri.002G142800	AGGAAAATAACGGAGCGCCA	GGTCGGTATTCGGGTACGAG
PtrGATA7	Potri.002G199800	AGGAGGAGGGCTCTTCTTGG	ATGGCAGCCTGTTCTTCCTC
PtrGATA8	Potri.003G082800	TGACAATAGGGTTGCTCTGGA	TGGACCTGTCAAATCACTTGC
PtrGATA9	Potri.003G174800	TTCCCAGCAGTGGAAACCTG	TTCAAGAACGGAGACTGGGC
PtrGATA10	Potri.003G213300	CGGTCGAACACTCCTCAACA	CAACTCCGGTTACTGCACCT
PtrGATA11	Potri.004G161500	GGTTCCCTCCAAGGCAAGAA	GGATGACGGTGTCTCGGTTT
PtrGATA12	Potri.004G211800	GATGATGGAGAAGACTGGGAGT	CAGCAGAGCTTGCCCAATGT
PtrGATA13	Potri.005G020500	TGCTCAGGTTGTTAGTGGCA	GCCATCAGAGAAAAGGCAGC
PtrGATA14	Potri.005G066100	GATGATGGAGAAGACTGGGAGT	CAGCAGAGCTTGCCCAATGT
PtrGATA15	Potri.005G117600	GTGAACTTTGTGTGCCGACG	AGGCTCTGTTAGGACCGACT
PtrGATA16	Potri.005G122700	GCAACAGCAACTTCGCCAAT	GGACCGTTTCTCCACAGAGG
PtrGATA17	Potri.005G152500	GTGGTGGTGTGGTTACGACT	CTGCTTGCACCTTTTCTGGG
PtrGATA18	Potri.005G152800	GTGGTCCATCTGGTCCAAGG	TCAATGGCAGTTCCAGAGTCC
PtrGATA19	Potri.006G229200	TCCCTTAACAGACGTGCCTT	TAGGCTGGAGTCATGGAGGG
PtrGATA20	Potri.006G237700	CCTCTTTCTCGGGCTGTGAG	TCGCTGCCAAGATGAACGTC
PtrGATA21	Potri.007G016600	TCCTAAGCAAGAAGCCCTTGA	ATCATCCGTCGGCACACAAA
PtrGATA22	Potri.007G024500	GCTCGCCGATGTGCTAATTG	ACCTGAGGCACTTGCATTGT
PtrGATA23	Potri.007G116600	AAACTTTGTGATGAAGGGGGC	GACCACGACGCATCATTGGA
PtrGATA24	Potri.007G116700	CCAAGCAATGGCATGCGTAA	TTGCCCACATAAGGCCACAA
PtrGATA25	Potri.008G038900	TTCGCCCACTTCGTCAATCA	CATCTCCACGGGACCTAACG
PtrGATA26	Potri.008G213900	CTGGTCCAAAGTCGCTGTGT	TCTCTGCCCAGTGCCAATAA
PtrGATA27	Potri.009G123400	GCTGGGTCGAACCAATCAGA	GTCTGAACCGTGTTGGTTGC
PtrGATA28	Potri.010G001300	TCTCTTATTGTTTTCCAGCAGGG	CTTCTTCTGTGGGCTCTCGG
PtrGATA29	Potri.010G223300	ATAGCGCCCTTTCCTTTCCT	TGTTTGAGTTTTGAAACAGGGAGG
PtrGATA30	Potri.010G251600	AGGATGCAAACGAGCACAGA	ACCGTTCACCACGCCATTAT
PtrGATA31	Potri.013G059600	CCAGCTCAGACCCTCCTTTG	GTGCACTCCCACTCCTGATT
PtrGATA32	Potri.014G058600	CCATCTCTCAGTCCCAACCG	TGCTCGGAAAGTCGGAGAAC
PtrGATA33	Potri.014G124400	GAGAGCACTTCATGGGTCGG	AGCCTGTTCTTCCTCTCCCA
PtrGATA34	Potri.017G042200	ACGAAGAATTGCCTCCCTGG	TGACGATGCAAATTGTCCTTTCT
PtrGATA35	Potri.017G042300	CGCAGGGTTCTGGACAAGAT	CGGAGTGGACTTTGAACCGA
PtrGATA36	Potri.018G044900	TCACTTAGGCAGCGAGCTTT	CCGAGCTTTCATGCCGGATA
PtrGATA37	Potri.018G053600	GCGGTTATTTCCGTTGAGGC	CTTGCACAACATGGCTCGTC
PtrGATA38	Potri.019G033000	TCCCAGCACCAACAAGTGAT	TCTTGCCTTTCCTGGGACTG
PtrGATA39	Potri.T158300	CACCAACTCCACGGACTCTT	AAAGCTCGCTGCCTAAGTGA

PtrUBQ	Potri.014G115100	AGACCTACACCAAGCCCAAGAAGAT	CCAGCACCGCACTCAGCATTAG

Table S2 The qPCR primers used for expression profiles of genes differentially expressed in the leaf, stem, and root of *oxPdGNC*, *crispr-GNC*, and wild type plants

Gene model ID	Primer-F (5'-3')	Primer-R (5'-3')
Potri.009G080600	CACAGACACTCCCTGTGGAC	TGTGCTTCCATAGCCTTCCG
Potri.004G053400	TCGGAAGGATCCCTTGGAGT	GTAGCCCCAGGTGACAAAGG
Potri.003G020400	TTTGAGGCTGGTCAAGGCAA	AACCAAAACGAGCATGGCAC
Potri.001G210000	TTGGGGTTTGCAGGGACTTT	GCCCACAGAACTGAGAAGGG
Potri.005G239300	AAGGGACCACTGGAGAACCT	TATGGGAACGATGACGGCAG
Potri.005G239200	ACGGCCACCAAACATCATCT	TTTCCTCATGGAGACACGGC
Potri.012G130600	CGGAGGGGTCAAGCCAAATA	GAAAGTCCCTGACCTCCAGC
Potri.018G091400	TCTGTCCTACCTGCCTCCTC	TGGGGAGCTTCCACAATGTC
Potri.006G239700	GTCGAGTTCGTCACCACCTT	GCCAATAGTCACTGCTGGGT
Potri.011G020900	GAAATGGTGGCCAGCAAAGG	CTGGTTTAGGGCATGGAGGG
Potri.013G087000	GAAAGCTCCAGACCTGCCTT	AATAGCCATAGCTGCGGGTC
Potri.001G278500	TTGCTGAGGCCCTTACAACC	TTACAGGCAGACAACGGGTC
Potri.007G144400	GTTTATTGCTTGAGCCCGAGG	TTAGCAACAAACACAGCACC
Potri.012G037200	TCCTCCTCCCCTAGCCAAAG	TTCCCAGGTGCAACAAACCT
Potri.017G139100	TGGATCCCGGACACTGGAAT	TTTCATGCTTGCGCTGGACT
Potri.001G162400	AAATGGTGCCTACACAGCCT	GACAACGGCAGCTATTGTGG
Potri.012G113500	TGCAGATGTGAAGGCCAAGT	CTGCTTACGCCGAGAGTGAA
Potri.001G070500	AGACATCTCCTTCCGAGACACTA	TGCAAGAGGGCATCGGAAATG
Potri.004G131600	CCAGCGTGTACTTGCTGAGA	CAAAAACAGGCGGCACCTAC
Potri.001G074000	AATTTGCTTTGCCTTGCCTG	TACTCGTCGGCAATGGCTTT
Potri.002G121500	GTTGGGAACTGTGCTTTGCT	TTCATGAGACAAAGGTGAGGC
Potri.014G021100	TTGTGGTTCCACATGCTCGT	GCAAGTGACCAAGCAAGGGT
Potri.009G086700	CCCCGAAACTGAGCCGATTA	GACCTCACCAGAACACTCCC
Potri.014G023000	GCATTCCTCGCTAGTGCCTTA	ATAAAGGGCCTAATGAAGTGGG
Potri.014G179400	GAAAGCGGGTGAGGGATTCA	ATGTCAGAAGAACACACGGT
Potri.001G348300	CACTCTTGCTTTCACCACGA	AGTCATAACTTGGCAGCCCT
Potri.009G097800	GGGTCATCGGTCTCTTCGTC	AGAAGCAAACAAGAGAGAGCATT

Table S3 The primers used in CRISPR/Cas9-mediated mutagenesis of *Populus GNC* gene.

Primer name	Sequence (5'-3')	Function
GNC_Mut_F1	TGTATGGCCTGCTTGGGTAA	Used for confirmation of target site of *Populus GNC* locus
GNC_Mut_R1	CCTCGTAGATGACGATGCCT	
GNC_Mut_F2	TATATCATGTATGGCCTGCT	
GNC_Mut_R2	TACCTTAGTGCTCCTCGTA	
GNC_Mut_F3	TCTGTGGCGACCGTACATCAT	
GNC_Mut_R3	TCAGCTGTGAATAAAGCCAC	
gRNA_GNC_F	attgGCGGACCACTCCTCCACAGC	Used for guide RNA construction
gRNA_GNC_R	aaacGCTGTGGAGGAGTGGTCCGC	

Table S4 The summary of GATA subfamilies in *Populus*, *Arabidopsis*, and rice. The atypical configuration in subfamily VI is 16 residues in the zinc-finger loop and the half zinc finger.

Structure	C-X_2-C-X_{18}-C-X_2-C				C-X_2-C-X_{20}-C-X_2-C		More than one zinc finger
Subfamily	I	II	IV	VII	III	V	VI
Protein domains	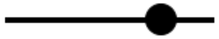	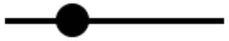	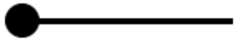	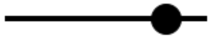	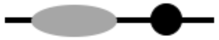	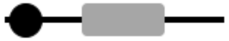	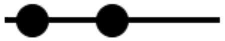
Gene structure	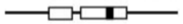	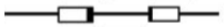	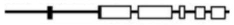	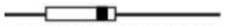	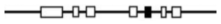	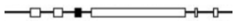	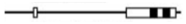
Description	Zinc finger at the C-terminal	Zinc finger is split into two exons	Zinc finger at the N-terminal	Only one exon	Zinc finger at the C-terminal	Zinc finger at the N-terminal	Including atypical configuration and standard C-X_2-C-X_{18}-C-X_2-C motif
Populus	Y	Y	Y	N	Y	N	N
Arabidopsis	Y	Y	Y	N	Y	N	N
Rice	Y	Y	N	Y	Y	Y	Y
